# Medicinal effects of *Ephedra foeminea* aqueous extracts: Metabolomic characterization, biological evaluation, and molecular docking

**DOI:** 10.1371/journal.pone.0328995

**Published:** 2025-07-24

**Authors:** Giulia Bennici, Inas Al Younis, Abeer Sharfalddin, Mutaz Akkawi, Fuad Al-Rimawi, Khaled Sawalha, Abdul-Hamid Emwas, Mariusz Jaremko

**Affiliations:** 1 Division of Biological and Environmental Sciences and Engineering (BESE), King Abdullah University of Science and Technology (KAUST), Thuwal, Saudi Arabia; 2 Department of Chemistry, Faculty of Science, King Abdul Aziz University, Jeddah, Saudi Arabia; 3 Biology Department, Faculty of Science and Technology, Al-Quds University, Jerusalem, Palestine; 4 Chemistry Department, Faculty of Science and Technology, Al-Quds University, Jerusalem, Palestine; 5 Core Lab of NMR, King Abdullah University of Science and Technology (KAUST), Thuwal, Saudi Arabia; Airlangga University Faculty of Medicine: Universitas Airlangga Fakultas Kedokteran, INDONESIA

## Abstract

*Ephedra foeminea* Forssk. is a medicinal plant traditionally used across various cultures and recognized for its historical significance in herbal medicine. It has been used as an herbal infusion to treat multiple respiratory diseases, headaches, and nasal congestion, as well as to prevent and manage breast cancer. In order to evaluate the validity of this ancestral knowledge, we aimed to investigate hot aqueous extracts of *E. foeminea* branches and fruits firstly by using untargeted metabolomic analysis, characterizing the chemical profiles of hot aqueous extracts from *E. foeminea* branches and fruits through the use of nuclear magnetic resonance, gas chromatography–mass spectrometry, and liquid chromatography–mass spectrometry. Subsequently, two *in vitro* studies were conducted to assess the anticancer and antioxidant potentials of the extracts. Antioxidant activity was evaluated using a 2,2-diphenyl-1-picrylhydrazyl assay and a total phenolic content assay. Anticancer activity was evaluated by assessing cytotoxicity using the MTT assay on MCF-7 (human breast cancer) and HeLa (cervical cancer) cell lines. Additionally, molecular docking was performed to explore the interactions between compounds identified in *E. foeminea* and selected cancer-related proteins, as well as the main protease of SARS-CoV-2. The results revealed that the branch extract exhibited superior antioxidant activity compared to the fruit extract, which was associated with a higher phenolic content in the branch extract (49.5 ± 0.7 mg GAE/g). The fruit extract exhibited greater cytotoxicity against MCF-7 cells, suggesting potential anticancer activity. Molecular docking analysis identified henryoside, guajavarin, and neohancoside as the most active compounds with anticancer and antiviral properties. These findings support the traditional use of *E. foeminea* Forssk. and highlight its potential as a source of bioactive compounds for further research into therapeutic applications.

## Introduction

Historically, disease treatment has relied on natural remedies, particularly medicinal plants [1–3]. Plants have long been recognized as valuable sources of bioactive compounds by traditional medical systems, including Traditional Chinese Medicine, Ayurveda, Unani, and various herbal practices [4–7]. Modern pharmacological research continues to validate many of these traditional remedies, identifying novel compounds with significant therapeutic potential, such as antimicrobial, anti-inflammatory, anticancer, and antioxidant activities [8–10]. However, with the emergence of germ theory and the rise of modern industrial chemistry, the use of medicinal plants has shifted away from experience-based traditional practices toward the development of synthetic medications—often derived from compounds isolated from plant sources (e.g., aspirin). Due to frequent toxicity, occasional severe side effects, and resistance associated with synthetic products, the use and exploration of natural pharmaceuticals are being revived [11,12].

The *Ephedra* genus (Ephedraceae), which includes *E. foeminea,* is among the oldest known groups of medicinal plants, it comprises 69 species, four subspecies, and two accepted varieties, all of which are widely distributed across arid and semi-arid regions of Asia, Europe, Northern Africa (Sahara), southwestern North America, and South America [13,14]. They are commonly utilized worldwide to treat various ailments [15]. In Arab regions, Ephedraceae species, particularly *Ephedra altissima*, have traditionally been used for medicinal purposes, including breast cancer treatment [13]. Using modern techniques, pharmaceutically active plant secondary metabolites have been isolated from soluble extracts from both aerial parts and roots of *Ephedra,* showing that the compositions and amounts of therapeutic components differ between species and elements of the plants [16].

Studies of this species led to the discovery in 1885 of ephedrine. The compound was later widely used as a nutritional supplement for weight loss and as a performance enhancer in sports. However, due to its effects on the sympathetic nervous system—including vasoconstriction and cardiac stimulation—the U.S. Food and Drug Administration banned ephedrine-containing supplements in 2004. Unlike other species in the *Ephedra* genus, which are widely known to contain the bioactive alkaloids ephedrine and pseudoephedrine, *E. foeminea* lacks these compounds, making it a valuable subject for research as it reduces concerns regarding toxicities, thus facilitating safer exploration for potential therapeutic applications and avoiding regulatory challenges [17]. *E. foeminea* (Arabic common name: Ala’andah علندة), with synonyms *E. campylopoda* and *E. fragilis* [18], is a shrubby plant with tiny leaves and multiple green branches, blooming during spring and summer months, with red ripened fruits appearing in October. *Ephedra foeminea* commonly grows in native habitats in the Mediterranean basin, including Greece, Turkey, Cyprus, Syria, Lebanon, Palestine, Egypt, but has also spread to Iraq and Saudi Arabia [19].

In traditional Asian medicine, *E. foeminea* is cherished for its preparation as a soothing infusion in hot water, a practice that highlights its cultural significance and health benefits [20]. However, high doses of the extract may be toxic, necessitating caution in its preparation and consumption. Contaminants—pollutants, metals, and bacteria—can pose health risks and compromise safety [21]. Recent studies using extracts from aerial parts of *E. foeminea* have shown bioactivity against cancer cell lines from the lung, liver, breast, colon, prostate, and bones [22,23]. This activity is assumed to be due to alkaloids, flavonoids, and sterols, all bioactive compounds [23,24]. For example, an *in vitro* study on the methanolic leaf extract of *E. foeminea* showed cytotoxic effects against various breast cancer cell lines, including evidence of apoptosis induction. Liquid chromatography–mass spectrometry (LC–MS) analysis identified 64 compounds with potential therapeutic value. Notable phytocompounds include ferulic acid, caffeic acid, and 7-Hydroxy-4-methylcoumarin, all recognized for their potential anticancer properties [17].

Extracts from *E. foeminea* are rich in total phenolic content (TPC), a key indicator of antioxidant potential. This property aligns with the reported anti-inflammatory, antibacterial, anticancer, wound healing, antidiabetic, and antiarthritic effects of *E. foeminea* [20,25,26].

Various analytical techniques, including gas chromatography–mass spectrometry (GC–MS), LC–MS, and nuclear magnetic resonance (NMR) spectroscopy, are used to analyze bioactive metabolites [27,28]. Each method has distinct advantages and limitations. GC–MS separates volatile compounds using gas chromatography and identifies them through mass spectrometry. Conversely, LC–MS is suited for non-volatile substances, utilizing liquid chromatography for separation and mass spectrometry for detection [29,30]. NMR spectroscopy provides detailed structural and quantitative information about metabolites. It is rapid, non-destructive, and highly reproducible, enabling repeated measurements to monitor metabolite flux over time [31–34]. However, NMR has limitations, such as low sensitivity and peak overlap, which can hinder the identification of low-abundance compounds [35,36]. Mass spectrometry overcomes these limitations and, when combined with chromatographic techniques, offers enhanced sensitivity and selectivity for metabolite profiling.

Therefore, the objective of this study is to profile the metabolic composition of *E. foeminea* aqueous extracts using untargeted metabolomics approaches, including NMR, LC–MS, and GC–MS, and to evaluate their potential antioxidant and anticancer activities. Additionally, molecular docking was performed using AutoDock Vina 4.0 to explore the *in vitro* findings and assess the binding efficiency of the identified compounds against cancer-related proteins and the SARS-CoV-2 main protease.

## Materials and methods

### Plant material

**Fig 1:**
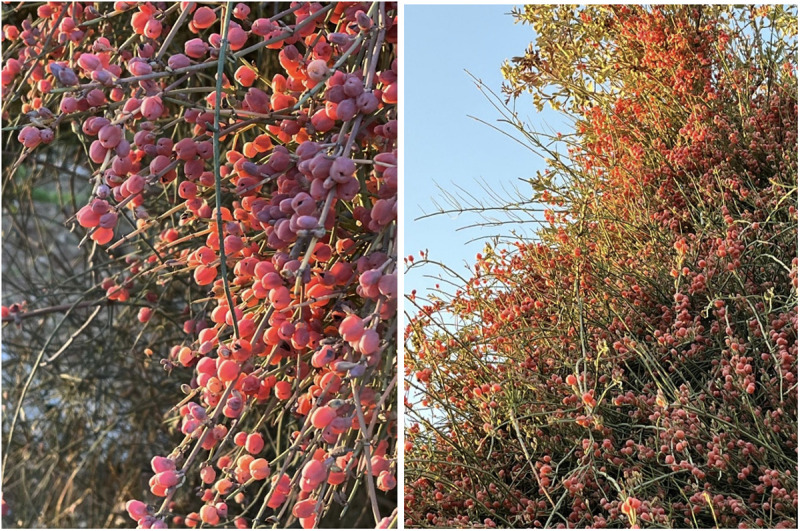
Images of *E. foeminea* (Arabic common name: Ala’andah) grown in its native habitat in the West Bank of Palestine.

*E. foeminea* was collected in 2022 from Baninaim in the West Bank, Palestine, and deposited at the Al-Quds University Herbarium under voucher reference number EPH-Pal-22. The specimens included green branches and red fruits (Fig 1), which were separated and air-dried in a shaded room at ambient temperature. The dried branches were ground into a fine powder using a Geno/Grinder^®^ 2010 homogenizer (SPEX SamplePrep, Metuchen, NJ, USA), while the fruits were powdered using a SPEX SamplePrep freezer/mill. The resulting powders were stored at −20°C.

### Samples preparation

Powdered *E. foeminea* branches and fruits were extracted using ultrapure water (Sigma–Aldrich). For each extraction, 200 mg of plant material was moistened with 2 mL of ultrapure water in an Eppendorf tube and incubated in a ThermoMixer^®^ (Eppendorf AG, Hamburg, Germany) at 80°C. Five replicates were prepared for each sample. The mixtures were then sonicated using a Fisherbrand^™^ FB-11203 shaker (Thermo Fisher Scientific, Waltham, MA, USA) at 80 kHz and 80°C. Subsequently, the samples were centrifuged at 15,000 rpm for 2 min at 4°C to separate the supernatant from the pellets. The resulting crude extracts were transferred to new tubes and stored at −20°C for further analysis.

### NMR analyses

Given the spectral complexity and limited availability of comprehensive reference databases for plant extracts, NMR was primarily used for metabolic fingerprinting rather than complete structural characterization.

#### Liquid-state NMR.

Each crude extract sample was retrieved from storage, and 1 mL was transferred to a fresh tube and vacuum-dried overnight using a CentriVap® vacuum concentrator (Labconco Corporation, Kansas City, MO, USA). Each dried extract was then dissolved in 2 mL Eppendorf tubes to a final concentration of 20 mg/mL using 600 μL of deuterium oxide (D_2_O, 99.9 atom% D; Sigma–Aldrich, St. Louis, MO, USA) containing 0.5 mM tetramethylsilane as an internal reference. The samples were vortexed, and 550 μL of the solution was transferred to 5 mm NMR tubes for ^1^H NMR analysis using an 800 MHz Bruker Avance III spectrometer (Bruker BioSpin, Billerica, MA, USA).

The ^1^H NMR spectra were recorded using 128 scans, a delay time of 5 s, and a temperature of 298 K, using the 90° pulse sequence (zgesgp) from the Bruker pulse library, combined with a water suppression pulse program. All liquid-state NMR spectra were recorded under the same conditions and parameters to ensure data comparability. Spectra analysis was conducted using TopSpin software (Bruker Daltonics, Germany), with tetramethylsilane at a concentration of 0.5 mM as the reference standard. Chemical shifts of all spectra were calibrated by setting the reference peak at 0.0 ppm. The phase was corrected manually, beginning with zero-order followed by first-order correction, while baseline correction was automatically applied using the “abs n” function in TopSpin. Spectra integration was then conducted using the integration command in TopSpin, based on the regions specified in the Results section.

#### Solid-state NMR.

The ^13^C solid-state NMR spectra were acquired using a 400 MHz Bruker Avance III spectrometer equipped with a magic angle spinning (MAS) probe optimized for proton detection. The system operated at 400 MHz and featured double variable temperature control to ensure thermal stability, with the capability for multiple nuclei detection. The plant powder was packed evenly into a 4-mm zirconia rotor and sealed with a Vespel cap. The solid-state NMR spectra were acquired at a 4 kHz MAS rotation rate using a cross-polarization pulse sequence from the Bruker pulse library, with 14,336 scans collected. Additional parameters were optimized according to previously established protocols [37]. The resulting spectra were then analyzed using Topspin software (Bruker Daltonics, Germany).

### GC-MS

A total of 200 μL of crude extract from each sample was collected into fresh 2 mL Eppendorf tubes. The samples were vacuum-dried using a CentriVap® vacuum concentrator (Labconco Corporation, Kansas City, MO, USA). The dried material was derivatized following the protocol of Salem *et al*. [38]. Briefly, 30 μL of methoxyamine hydrochloride (15 mg/mL in pyridine) was added to each sample and mixed at 600 rpm (37°C for 90 min). Subsequently, 50 μL of O-bis (trimethyl-silyl)trifluoroacetamide (Sigma–Aldrich, USA), spiked with a 10 µg/mL C_7_–C_40_ saturated alkanes standard solution (Supelco, 49452-U), was added to each sample and mixed at 600 rpm (37°C for 30 min) to form trimethylsilyl derivatives. The samples were then centrifuged at 15,000 rpm for 3 min to allow pellet precipitation. A pooled quality control sample was prepared by mixing aliquots from all extracts. Finally, 30 μL of the resulting supernatant was transferred into amber GC vials containing fixed inserts for analysis. To minimize bias, the sample vials were analyzed in a randomized order.

Metabolite analysis was performed using a Thermo Scientific^™^ Orbitrap^™^ Exploris GC 240 mass spectrometer (maximum resolving power: 240,000 at m/z 200 FWHM). The samples were introduced using a Thermo Scientific^™^ TriPlus™ RSH autosampler. Gas-phase chemical components were separated using a Thermo Scientific^™^ TRACE^™^ 1310 Gas Chromatograph fitted with a Thermo Scientific^™^ TraceGOLD^™^ TG-5SilMS column (30 m × 0.25 mm id × 0.25 μm film thickness).

The Orbitrap^™^ Exploris GC 240 was tuned and calibrated using perfluorotributylamine to achieve a mass accuracy <1.0 ppm. The mass spectrometer operated in electron ionization mode, with the ion source set to 300°C. The electron energy was 70 eV, and the emission current was 50 µA. Data acquisition was performed in full-scan mode over a mass range of 35–700 Da, with a resolving power of 60,000 (FWHM at m/z 200). The automatic gain control target was set to “standard,” and the maximum injection time was set to “auto” mode. The data acquisition was lock-mass corrected using the GC column bleed siloxane mass at m/z 207. A 1-µL sample was injected into a single-taper gooseneck liner containing glass wool (Thermo Scientific^™^ Liner GOLD) with a 10-µL syringe. The inlet mode was set to split mode, with a 40 mL/min split flow rate and a 5 mL/min purge flow. Helium was used as the carrier gas at a constant flow rate of 1.2 mL/min. The oven temperature was initially held at 70°C for 2 min and then increased to 220°C at 8°C/min, followed by an increase to 325°C at 16°C/min, where it was maintained for 10 min.

The raw data obtained from the GC–MS analysis were processed using Compound Discoverer 3.3 software (Thermo Scientific, USA). An untargeted metabolomics workflow incorporating multivariate statistical analyses—including principal component analysis and volcano plots—was applied to identify significant features based on their m/z and retention times. Chromatographic peaks were then deconvoluted, aligned, filtered, and putatively identified using a mass spectral library match, including the NIST20 Nominal Mass Library and the Orbitrap GC–MS HRAM Metabolomics Library. Retention indices were calculated using the Kovats method based on a homologous series of n-alkanes (C_7_–C_40_) analyzed under the same chromatographic conditions. Only annotations with calculated retention indices (RI) exhibiting an RI delta of <50 and a similarity index (SI) >600 were retained. Spectra were manually compared against online databases to verify the matches. Identified metabolite names were standardized using the RefMet nomenclature (The Metabolomics Workbench) and classified using the RefMet conversion tool (https://www.metabolomicsworkbench.org/databases/refmet/name_to_refmet_form.php, accessed on 11 November 2024).

### UHPLC-MS (timsTOF)

The metabolic profile of *E. foeminea* extracts was analyzed through ultra-high-pressure liquid chromatography coupled with mass spectrometry (UHPLC–MS) on a timsTOF Pro system (Bruker, Germany). This platform integrates the LC–MS with trapped ion mobility spectrometry (TIMS), enhancing peak capacity and improving confidence in compound characterization. The TIMS device, positioned at the front of a quadrupole time-of-flight (QTOF) mass spectrometer, allows ions to accumulate for a specific period before being released for MS analysis [39]. This instrument can generate four-dimensional descriptors—retention time, m/z, MS/MS, and collision cross-section—enabling comprehensive compound identification.

*E. foeminea* crude extracts (200 μL) from branch and fruit samples were transferred into fresh 2 mL Eppendorf tubes. A pooled quality control sample was prepared by mixing aliquots from various extracts. Both the pooled samples and a blank were vacuum-dried using the CentriVap® vacuum concentrator (Labconco Corporation, Kansas City, MO, USA). The dried samples were then resuspended in 250 μL of a methanol:water (8:2 v/v) solution, vortexed, and centrifuged at 15,000 rpm (4°C for 3 min). Subsequently, 50 μL of each sample was transferred into amber LC vials for analysis. Electrospray ionization (ESI) was applied in positive (ESI+) and negative (ESI−) modes.

The samples were injected into a C18 column (Acquity CSH, 100 × 2.1 mm, 1.7 μm particle size). The gradient elution program was as follows: 0–1.5 min, 1% solvent B; 15 min, 100% B; 17 min, 100% B; 17.5 min, 1% B; and 21 min, 1% B, with the flow rate maintained at 0.4 mL/min. The column temperature was set to 35°C, and the injection volume was 10 µL. Data acquisition was performed in parallel accumulation–serial fragmentation mode over an m/z range of 20–1200. The collision energy for MS/MS analysis was stepped between 20 and 40 eV.

Following data acquisition, the raw data from the UHPLC–MS were processed and annotated using MetaboScape software (Bruker Daltonics, Germany).

### Antioxidant analyses

A 2,2-diphenyl-1-picrylhydrazyl (DPPH) assay was employed to assess the antioxidant activity of *E. foeminea* extracts. A methanolic dilution of DPPH was prepared at a final concentration of 0.25 mM and stored in the dark. Ascorbic acid was used as the reference standard, and a calibration curve was generated using serial dilutions of ascorbic acid in methanol ranging from 5 to 30 μg/mL. Crude extracts from the branches and fruits of *E. foeminea* were vacuum-dried, and the resulting pellets were resuspended in water. Sample concentrations ranged from 1500 to 200 μg/mL. Methanol served as the control and was pipetted into a 96-well plate with the samples and standard. DPPH solution was then added to each well, and the plate was incubated in the dark for 30 min. Absorbance was measured at 517 nm using a Cytation^™^ 5 cell imaging multimode reader (BioTek Instruments Inc., USA).

The percentage of inhibition was calculated using the following formula:


$$\;\%inhibition=\frac{Absorbance\;of\;control\;-\;Absorbance\;of\;samples}{Absorbance\;of\;control}\;\times \;100$$

The IC_50_ for each extract was determined from a linear regression curve of % radical scavenging against extract concentrations (μg/mL), using the equation y = mx + c, where y was set to 50.

### Total phenolic content

TPC was determined using the Folin–Ciocalteu reagent assay. The Folin–Ciocalteu reagent (Sigma–Aldrich) was diluted in water to a final concentration of 10%. Gallic acid, diluted in methanol at varying concentrations, was used as the standard. Crude extracts of the plants were diluted in water to a concentration of 1000 ppm. Aliquots of the samples, prepared in triplicate, along with the standard solutions and a blank (water), were dispensed into a 96-well plate. The diluted Folin–Ciocalteu reagent (10%) was added to each well, and the plate was incubated in the dark for 10 min. Subsequently, a 7.5% Na_2_CO_3_ reagent, previously dissolved in water, was added, followed by an additional 30-min incubation period. Absorbance was measured at 750 nm using a Cytation^™^ 5 cell imaging multimode reader (BioTek Instruments Inc., USA). The calibration curve was generated using the gallic acid standard diluted in methanol at 25–175 ppm concentrations. The equation derived from the standard curve was used to extrapolate the results, which were expressed as milligrams of gallic acid equivalent (GAE) per gram of dried sample. Data are presented as the mean of three independent determinations ± standard deviation (SD) [40,41].

### Anticancer assay (MTT)

Cell viability was assessed based on the enzymatic reduction of the tetrazolium dye MTT to formazan. To evaluate the anticancer activity of the crude extracts of *E. foeminea* branches and fruits, two cancer cell lines were used: MCF-7 (a human breast cancer cell line) and HeLa (a cervical cancer cell line). HEK295 cells (human embryonic kidney cells) were used as a noncancerous control for cytotoxicity. The three cell lines were cultured in Dulbecco’s Modified Eagle’s Medium, supplemented with 0.2% sodium bicarbonate and 10% fetal bovine serum. The cells were maintained at 37°C in a humidified atmosphere of 5% CO_2_. Cell viability was evaluated using the trypan blue dye exclusion method, as previously described [42,43]. Only cell batches exhibiting viability >98% were used in this study. The MTT assay was then performed, following established protocols [44,45] to determine the cytotoxic effects of the plant extracts.

The crude extracts were vacuum-dried, and the resulting pellets were resuspended in water. Various concentrations of the resuspended samples were prepared. Briefly, 10,000 cells from each cell line were seeded into 96-well culture plates and allowed to adhere for 24 h in a CO_2_ incubator at 37°C. Following adherence, the cells were exposed to different concentrations of each prepared extract. Subsequently, MTT (5 mg/mL in PBS) was added to each well (10 μL per 100 μL of cell suspension), and the plates were incubated for an additional 4 h in the CO_2_ incubator. The supernatant was discarded, and 200 μL of DMSO was added to each well and mixed gently. Absorbance was measured at 550 nm using an Evolution 201 microplate reader (Thermo Scientific, Waltham, MA, USA). Untreated control wells were included and processed under the same conditions. All absorbance values were corrected for background interference.

The survival percentage was calculated as (ODc/ODt) × 100%, where ODc denotes the optical density of untreated cells and ODt represents the optical density of wells treated with the test compound. Survival curves for each cancer cell line and the standard cell line were constructed by plotting the relationship between cell survival and compound concentrations. The IC_50_ was determined using the dose-response curve generated with GraphPad Prism software (San Diego, CA, USA) for all cell cultures.

### Molecular docking

Molecular docking, a computational method, was performed to assess the potential bioactivity of chemical molecules and their effectiveness against cancer and viral proteins. Among the annotated molecules identified through the MS, some compounds (S1 File) were selected based on their distinct functional groups (alkaloids and flavonoids) and their established efficacy as bioactive molecules, as previously reported [46–52].

The selected molecules were retrieved from PubChem in a three-dimensional (3D) SDF format. PyMol [53] was used to convert the structures to Protein Data Bank (PDB) format for compatibility with AutoDock 1.5.7 software. The following steps were then performed: Gasteiger charges were added, nonpolar hydrogen atoms were merged, rotational interactions were identified, and the files were saved in PDBQT format.

As previously noted, three target proteins were selected for this study: two cancer-related proteins (PBD IDs: 1HK7 and 6I2I), and the main protease of SARS-CoV-2 (PDB ID: 6LU7), included due to the traditional use of *E. foeminea* in treating respiratory ailments [42,54]. All crystal structures were retrieved from the PDB. The Discovery Studio 2024 Visualizer was used to prepare the macromolecular structures by removing water molecules, ligand compounds, and other nonessential components, after which the cleaned structures were saved in PDB format. Using AutoDock 1.5.7, polar hydrogen atoms and Gasteiger and Kollman charges were added to the prepared files, which were then saved in PDBQT format for molecular docking.

All selected *E. foeminea* compounds were docked against the target proteins using AutoDock Vina 4.0. For each protein, a 3D grid box was generated to encompass the entire receptor surface, allowing the ligands to interact with the amino acids in the active site. The grid box dimensions (X, Y, Z) were adjusted with a spacing of 0.3 Å and saved for subsequent analysis. The molecular docking study was performed following the Lamarckian Genetic Algorithm protocol. The docking results were visualized using Discovery Studio 2024 to analyze ligand–receptor interactions in two-dimensional (2D) and 3D formats.

## Results

### Metabolomic profile of E. foeminea extracts

#### Liquid-state NMR.

**Fig 2:**
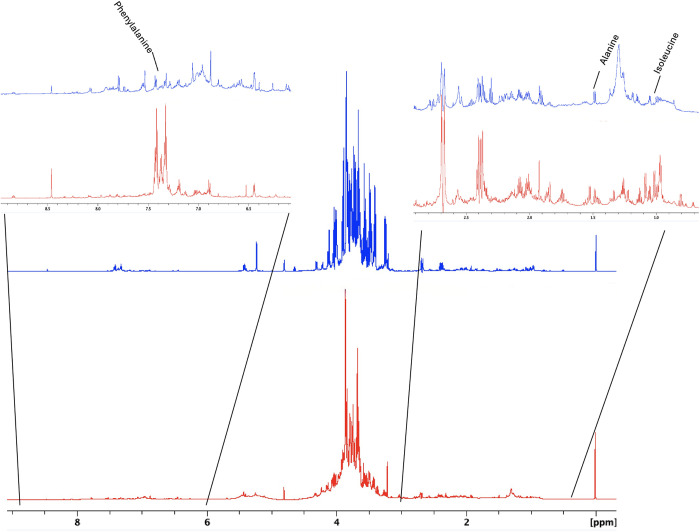
Representative 1D ^1^H NMR spectra of E. foeminea branches (red) and fruits (blue). The upper left inset highlights the aromatic region (6.7–9.5 ppm), and the upper right inset highlights the methylene region (1.5–3 ppm).

**Fig 3:**
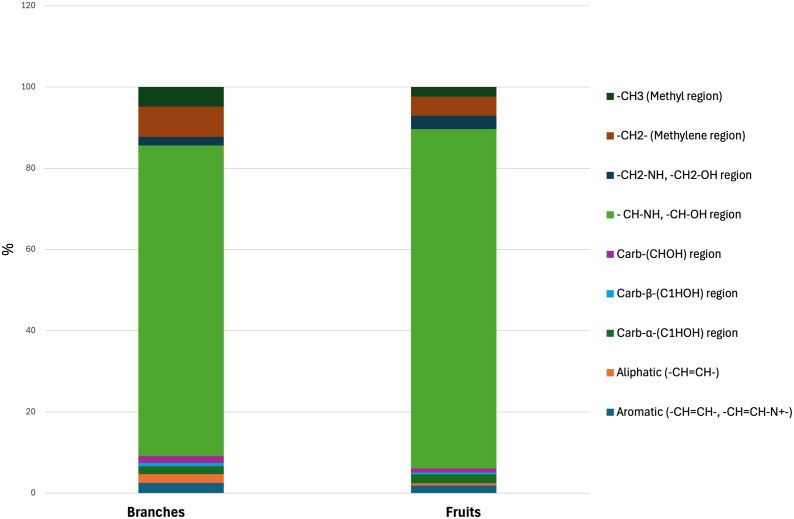
Graphical representation of the mean ratios of integrated regions from the NMR spectra of E. foeminea branch and fruit extracts.

In medicinal plant and food research, NMR-based metabolomics is widely regarded as the preferred methodology for metabolite analysis, with ^1^H NMR spectroscopy being the primary instrument employed. This method offers detailed insights into the intricate metabolic profiles of various substances, thereby enhancing our understanding of potential therapeutic properties [55]. In this study, one-dimensional (1D) ^1^H NMR spectra were obtained using an 800 MHz Bruker Avance III NMR spectrometer (Fig 2). Spectral data were analyzed using TopSpin software and segmented into nine chemical shift regions (ppm), each corresponding to specific functional groups, as shown in Table 1. Each NMR spectrum was integrated, and the percentage of each integrated region was calculated to estimate the relative concentrations of analytes in different parts of the plant (Table 2; Fig 3). In the branch extract, polysaccharides were the most abundant, accounting for 76% of the total spectral area. The aromatic and aliphatic regions contributed 2.6% and 2.1%, respectively (Table 2; Fig 3). A similar distribution was observed in the fruit extract, where polysaccharides constituted 83%, followed by the aromatic region at 1.88%, and the aliphatic region at 0.5%. Given the inherent complexity and signal overlap in the NMR spectra of plant extracts, full structural characterization of all metabolites was not feasible. Therefore, Chenomx Profiler software (Chenomx Suite 9.0, Edmonton, AB, Canada) was used to highlight and tentatively assign selected compounds—such as amino acids and polysaccharides—within the spectra (Fig 2; Figs S1–S6 in S1 File). These assignments were based on chemical shift comparisons. Table in S1 File presents the chemical shifts (ppm), multiplicity, and coupling constants (Hz) of the reported compounds.

**Table 1:** Functional groups and their relative chemical shifts (ppm) obtained from ^1^H NMR spectra.

Functional Groups	^1^H Chem. Shifts (ppm)
-CH_3_ (Methyl region)	0.70–1.50
-CH_2_ (Methylene region)	1.50–3.0
-CH_2_ -NH, -CH_2_ -OH	3.0–3.30
-CH -NH, -CH -OH	3.30–4.25
Carb-(CHOH)	4.25–4.40
Carb-β-(C1HOH)	4.40–4.65
Carb-α-(C1HOH) region	5.16–5.42
Aliphatic (-CH = CH-)	5.50–6.68
Aromatic (-CH = CH-, -CH = CH -N + -)	6.70–9.50

**Table 2:** Relative proportion of compounds in the E. foeminea branch and fruit extracts based on integrated ^1^H NMR spectra regions. Results are based on the mean of three replicates ± SD.

Region	% Branches	% Fruits
Aromatic (-CH = CH-, -CH = CH-N + -)	2.55 ± 0.72	1.88 ± 0.86
Aliphatic (-CH = CH-)	2.10 ± 0.92	0.5 ± 0.27
Carb-α-(C1HOH) region	1.99 ± 1.49	2.23 ± 0.29
Carb-β-(C1HOH) region	0.78 ± 0.30	0.44 ± 0.39
Carb-(CHOH) region	1.74 ± 0.64	1.07 ± 0.39
- CH-NH, -CH-OH region	76.45 ± 19.55	83.50 ± 7.35
-CH_2_-NH, -CH_2_-OH region	2.14 ± 0.54	3.31 ± 0.63
-CH_2_- (Methylene region)	7.43 ± 1.60	4.75 ± 1.35
-CH_3_ (Methyl region)	4.82 ± 0.97	2.33 ± 0.50

A comparison of the integrated peak percentages from the branch and fruit extracts was performed to identify which plant contained higher levels of bioactive metabolites. As shown in Fig 3, the branch extract exhibited higher metabolite concentrations across all regions except the -CH_2_–NH and -CH_2_–OH region and the Carb-α-(C1HOH) region or anomeric region. Notably, the branch extract showed a higher intensity in the aromatic, substituted aromatic, and phenolic regions.

#### Solid-state NMR.

**Fig 4:**
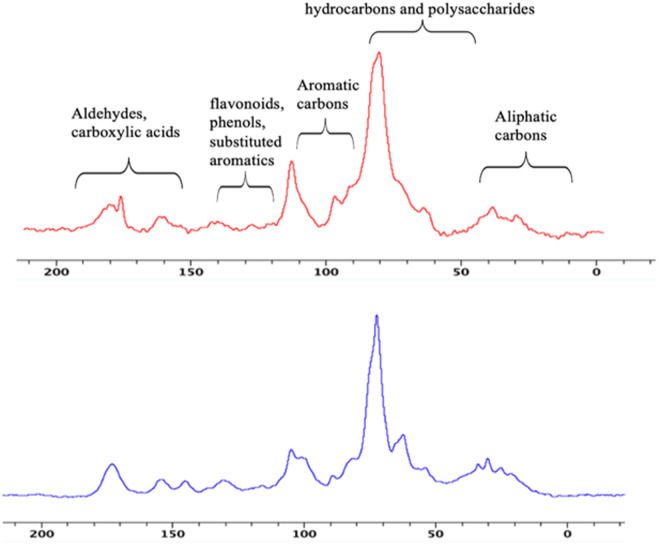
1D ^13^C solid-state NMR spectra of E. foeminea branches (red) and fruits (blue).

It is well established that solvents dissolve metabolites selectively, and no single solvent can extract all metabolites simultaneously. Therefore, solid-state NMR serves as a powerful tool for analyzing the chemical composition of plant materials in their native states. Solid-state ^13^C NMR spectroscopy was performed using a 400 MHz Bruker Avance III to complement and validate the liquid-state NMR results. The spectra obtained from the powdered branches and fruits are presented in Fig 4. These spectra were segmented into five regions corresponding to different functional groups: aliphatic carbons (10–45 ppm); hydrocarbons and polysaccharides (45–90 ppm); aromatic carbons (90–110 ppm); flavonoids, phenols, and substituted aromatics (120–140 ppm); and aldehydes and carboxylic acids (150–190 ppm) (Fig 4).

**Fig 5:**
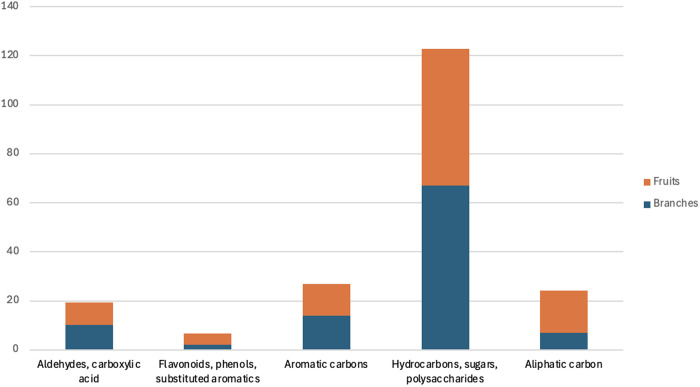
Percentage ratios of integrated regions from ^13^C solid-state NMR spectra of E. foeminea branch and fruit powders. Data are based on a single measurement performed for chemical fingerprinting purposes; therefore, statistical analysis was not applicable.

The solid-state NMR results, including the relative proportions of different functional groups, are presented in Fig 5. The region corresponding to hydrocarbons and polysaccharides (45–90 ppm) was the most abundant, consistent with the findings from the 1D ^1^H liquid-state NMR analysis. The regions associated with flavonoids, phenols, and substituted aromatics, as well as aliphatic carbons, showed higher intensities in the fruit samples than in the branches. This observation indicates that the extraction method was effective in extracting all bioactive compounds from the fruits. The fruits—red, fleshy, cone-like structures—contain seeds with a higher oil content, which likely renders them more hydrophobic than the branches.

#### GC-MS and UHPLC-MS(timsTOF).

To further characterize the metabolic profile of E. foeminea extracts, GC–MS and UHPLC–MS (timsTOF) analyses were conducted on the branch and fruit extracts. A total of 232 compounds were identified in the fruit extract and 117 in the branch extract (Tables in S1 File). The identified compounds were then annotated and classified. A summary of the phytochemicals identified through GC–MS and UHPLC–MS (timsTOF) is presented in Table 3.

**Table 3:** Summary of the main identified phytochemical components of E. foeminea branches and fruit extract, and their main class and sub-class according to the different analytical methods.

Phytochemicals Class	Phytochemicals Name		Method	Plant component
Isoprenoids	(a) C15 isoprenoids	Alantolactone	UHPLC-MS(timsTOF) (+)	Branches
Phenols	(a) monophenols	Henryoside	UHPLC-MS(timsTOF)(-)	Fruits
				
Phenylpropanoids	(a) Cinnamic acids	Ferulic acid	GC-MS	Fruits
		Mandelic acid	GC-MS	Fruits
		Cis-Melilotoside	UHPLC-MS(timsTOF)(-)	Fruits
		Neohancoside D	UHPLC-MS(timsTOF)(-)	Fruits
Flavonoids	(a) Flavonols	Epicatechin	UHPLC-MS(timsTOF) (+)	Fruits
		Guajavarin	UHPLC-MS(timsTOF) (+)	Fruits
		Kaempferol	UHPLC-MS(timsTOF) (+)	Fruits
		Kaempferol 3-glucoside-7-glucuronide	UHPLC-MS(timsTOF)(-)	Fruits
		Fisetinidol-4beta-ol	UHPLC-MS(timsTOF)(-)	Fruits
	(b) Rotenoids	3-O-demethylamorphigenin	UHPLC-MS(timsTOF)(-)	Fruits
				
Alkaloids	(a) Tryptophan alkaloids	Norharmane	UHPLC-MS(timsTOF) (+)	Fruits
		4-Formyl indole	UHPLC-MS(timsTOF) (+)	Fruits
	(b) Pyridine alkaloids	Trigonelline	UHPLC-MS(timsTOF) (+)	Fruits
				
	(c) Anthranilic acid alkaloids	6-Methylquinoline	UHPLC-MS(timsTOF) (+)	Branches
	(e) Lysine alkaloids	Piperine	UHPLC-MS(timsTOF) (+)	Branches
Benezenoids	(a) Hydroxybenzoic acids	3-Hydroxybenzoic acid	GC-MS	Fruits
		Benzoic acid	GC-MS	Fruits
		Vanillic acid	GC-MS	Fruits
		Gallic acid	GC-MS	Fruits
		3,4-Dihydroxybenzoic acid	UHPLC-MS(timsTOF) (-)	Fruits
	(b) Chatecols	Pyrocatechol	GC-MS	Fruits
Terpenoids	(a) Diterpene	Galdosol	UHPLC-MS(timsTOF) (-)	Branches

### Antioxidant activity

#### DPPH.

**Fig 6:**
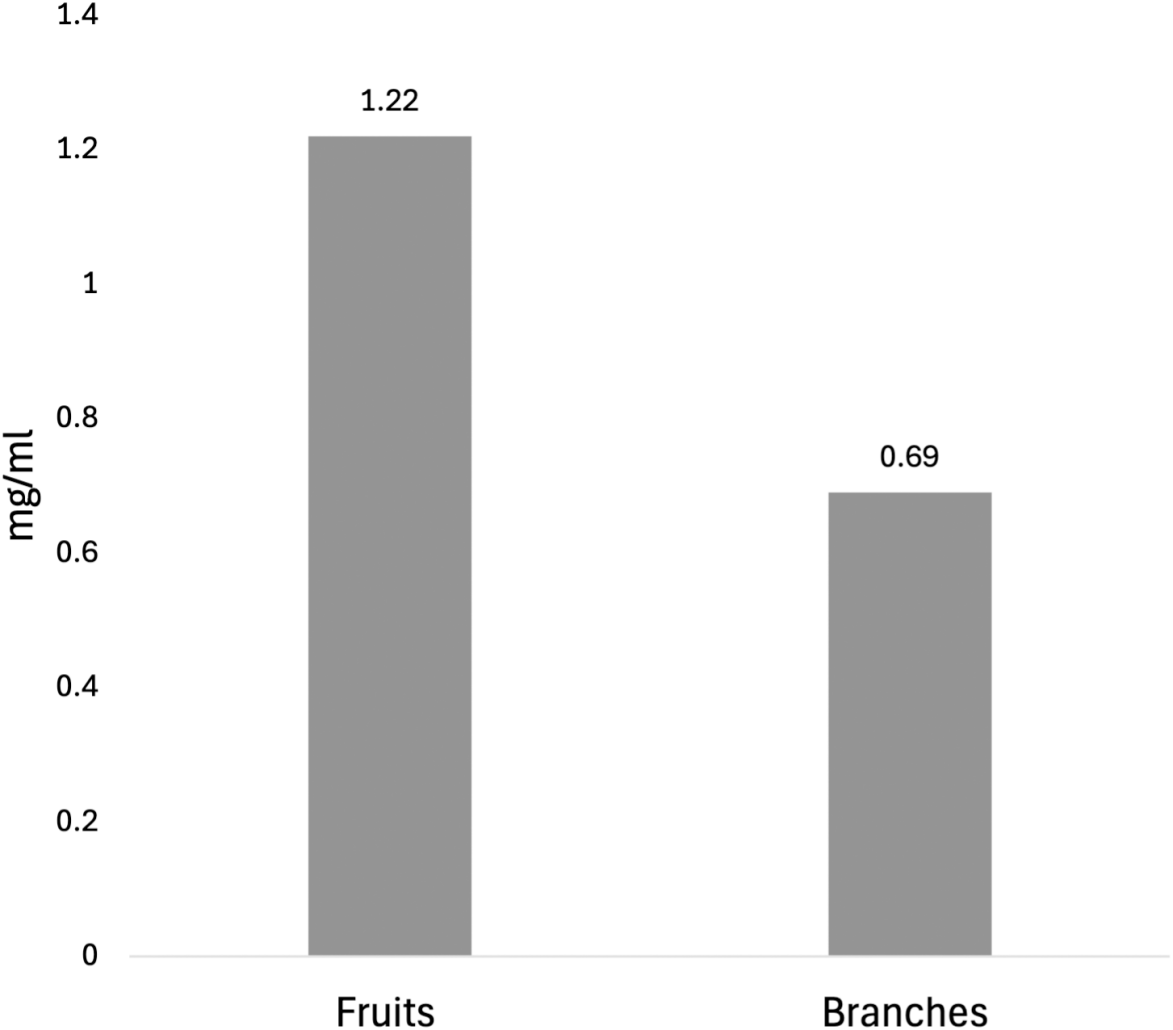
Percentage of DPPH scavenging activity of E. foeminea branch and fruit extracts.

The DPPH assay was used to assess the antioxidant activity of crude extracts from E. foeminea branches and fruits. Different extract concentrations (1500–100 μg/mL) were tested, and calibration curves were constructed to calculate the IC_50_ values. Ascorbic acid was used as a positive control, with concentrations ranging from 5 to 30 g/mL. The IC_50_ value for the branch extract was 0.69 ± 0.08 mg/ml. Conversely, the fruit extract had an IC_50_ value of 1.22 ± 0.1 mg/mL (Fig 6), calculated from the equation of the standard curve y=2.26x+19.30, with an R^2 ^= 0.99.

#### Total phenolic content (TPC).

**Fig 7:**
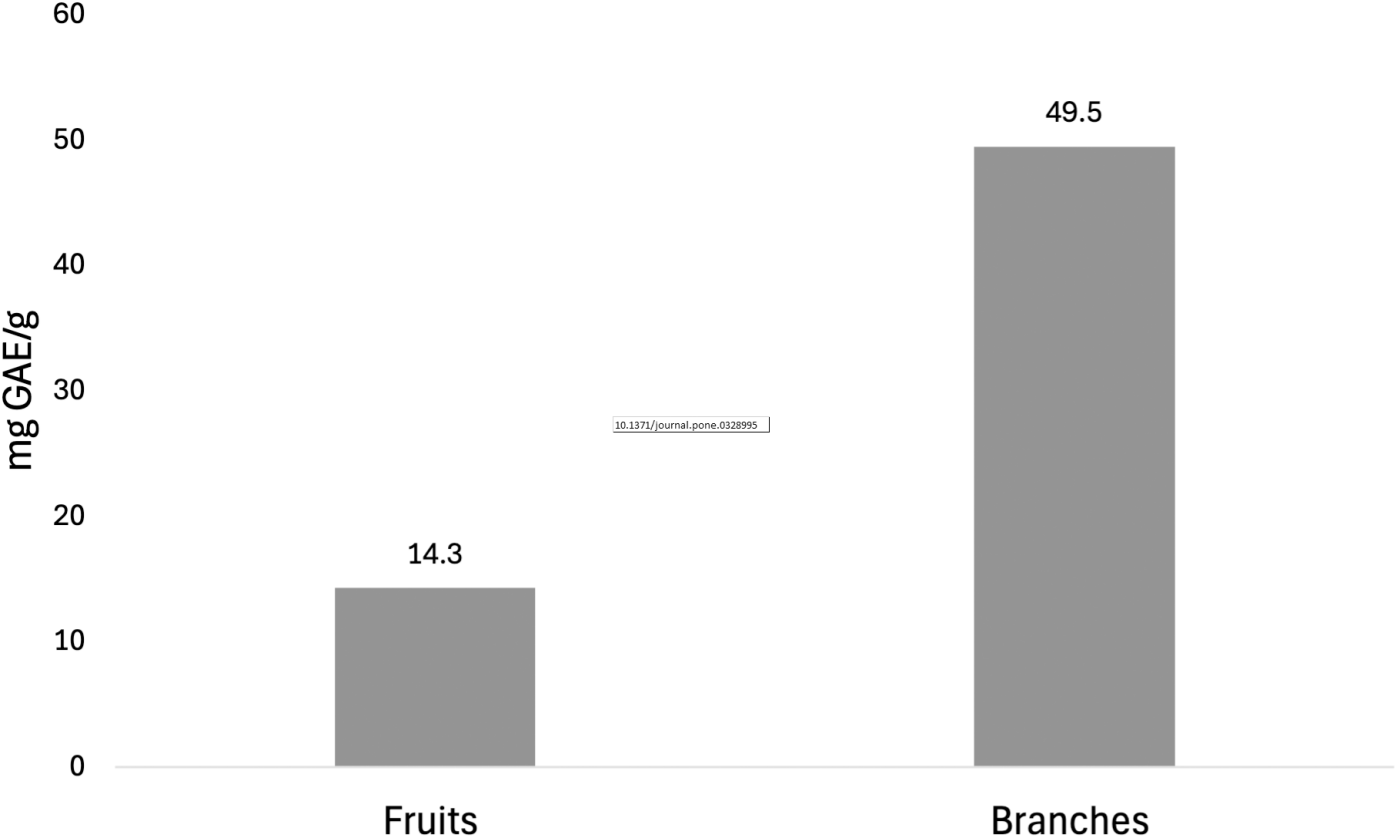
Total phenolic content of E. foeminea branch and fruit extracts.

Aromatic and substituted aromatic compounds, such as phenols, flavonoids, and alkaloids, are recognized as bioactive constituents. Consequently, it is essential to quantify total phenolic compounds and examine specific metabolite classes, including flavonoids and alkaloids. Crude extracts were analyzed for their TPC using the Folin–Ciocolteau assay. This assay was conducted on fruit and branch extracts at 1000 μg/mL concentration, with gallic acid serving as the reference. TPC was expressed in milligrams of GAE per gram of dry extract, using the following formula:


$$GAE=c\;x\frac{V}{m}$$

where c is the sample concentration derived from the linear regression, V is the initial volume of the extract, and m is the initial mass of the crude dry extract. The TPC of the branch extract was estimated at 49.5 ± 0.7 mg GAE/g of dry extract, whereas the fruit extract yielded 14.3 ± 0.4 mg GAE/g of dry extract (Fig 7). These results are consistent with the DPPH assay results: higher IC_50_ values in the DPPH assay correspond to a lower TPC. The values were calculated using the gallic acid standard curve y=0.0044x+0.0329.

### Anticancer assay

**Fig 8:**
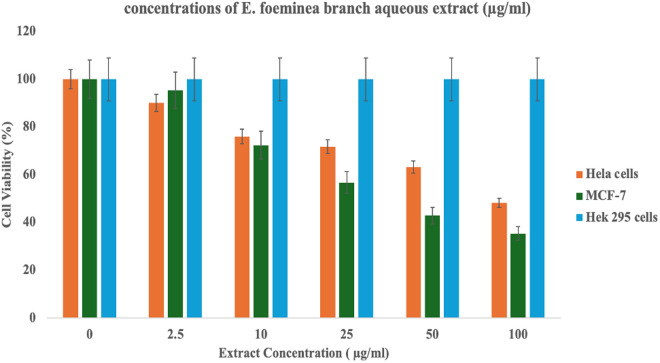
Viability of HeLa, MCF-7, and Hek 295 cell lines treated with varying concentrations of E. foeminea branch aqueous extract.

**Fig 9:**
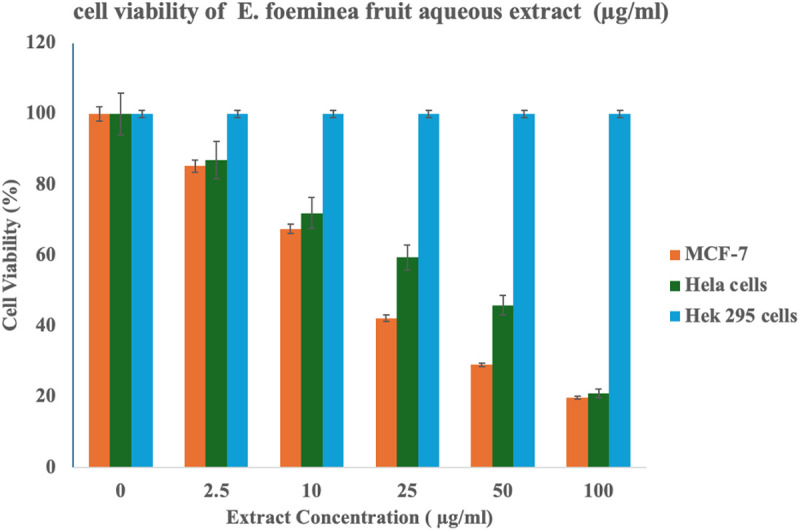
Viability of HeLa, MCF-7, and Hek 295 cell lines treated with varying concentrations of E. foeminea fruit aqueous extract.

To evaluate the antiproliferative activity of E. foeminea, samples of fruit and branch aqueous extract were tested for their effect on tumor cells using two cancer cell lines—MCF-7 (a human breast cancer cell line) and HeLa (a cervical cancer cell line). HEK295, a human embryonic kidney cell line, was used as a control to assess the selectivity and potential cytotoxicity of the compounds. The cell line viability results are presented in Figs 8 and 9, corresponding to the branch and fruit aqueous extracts, respectively, across varying concentrations. Both extracts exhibited antiproliferative activity, with a more pronounced effect observed against MCF-7 cells. Notably, the fruit extract exhibited significantly greater efficacy, as shown in Table 4, which presents the IC_50_ values of the extracts against HeLa and MCF-7 cell lines.

**Table 4:** IC_50_ values of E. foeminea fruit and branch aqueous extract against MCF-7 and HeLa cell lines expressed in µg/mL.

Compound	MCF-7 cells IC_50_ (µg/ml)	Hela cells IC_50_ (µg/ml)
*E. foeminea* fruits aqueous extract	23.6 ± 3.0	57 ± 3
*E. foeminea* branches aqueous extract	33 ± 2	67 ± 3

IC₅₀ activity scale: 1–10 (very strong), 11–20 (strong), 21–50 (moderate), 51–100 (weak), > 100 (noncytotoxic).

Results from the analytical profiling of the plants revealed that several compounds previously reported to possess anticancer activity were detected in the E. foeminea extract. Table 5 shows a summary of these phytochemicals, along with their reported anticancer activity.

**Table 5:** Summary of tentatively identified phytochemicals in E. foeminea fruits and branch extracts and their reported anticancer activities.

			GC-MS			UHPLC-MS (timsTOF)				
	No.	Compound name	RT (min)	Calc. RI	Delta RI	RT (min)	Delta m/z (ppm)	m/z	Adduct	Ref.
Fruits	1	Benzoic acid	8.188	1222	23	−	−	−	−	[56]
	2	Protocatechuic acid	17.196	1813	16	−	−	−	−	[57]
	3	Pyrocatechol	9.327	1288	17	−	−	−	−	[58]
	4	Succinic acid	11.521	1417	8	−	−	−	−	[59,60]
	5	Trigonelline	−	−	−	0.65	1.647	138.05518	[M+H]+	[61,62]
	6	Norharmane	−	−	−	5.79	0.482	169.07594	[M+H]+	[63]
	7	Epicatechin	−	−	−	6.55	0.624	291.0865	[M+H]+	[64]
	8	Guajavarin	−	−	−	8.21	0.962	435.0926	[M+H]+	[65]
	9	Kaempferol	−	−	−	8.73	1.821	287.05554	[M+H]+	[66]
	10	3,4-Dihydroxybenzoic acid	−	−	−	5.21	1.018	153.01949	[M-H]–	[67]
	11	Djalonensone	−	−	−	11.96	0.536	271.06134	[M-H]–	[68]
	12	Cis-Melilotoside	−	−	−	6.58	1.092	325.09325	[M-H]–	[69]
	13	Neohancoside D	−	−	−	6.67	0.369	547.16664	[M-H]–	[70,71]
	14	Henryoside	−	−	−	9.07	0.123	583.16692	[M-H]–	[72]
Branches	15	Succinic acid	9.724	1312	5	−	−	−	−	
	16	Caprylic acid	8.113	1219	15	−	−	−	−	[73]
	17	6-Methylquinoline	−	−	−	2.21	1.381	144.08097	[M+H]+	[74]
	18	Caffeine	−	−	−	6.57	0.851	195.08782	[M+H]+	[75]
	19	Alantolactone	−	−	−	11.10	0.258	233.15355	[M+H]+	[76]
	20	Nonanedioic acid	−	−	−	8.52	0.25	187.0976	[M-H]–	[77]
	21	Galdosol	−	−	−	11.03	0.391	343.15496	[M-H]–	[78]

Data were obtained using GC–MS and UHPLC–MS (timsTOF). RT, retention time; RI, retention index; m/z, mass-to-charge ratio; Δm/z, mass error in parts per million (ppm); [M + H]^+^, protonated ion; [M–H]^−^, deprotonated ion.

### Molecular docking

**Fig 10:**
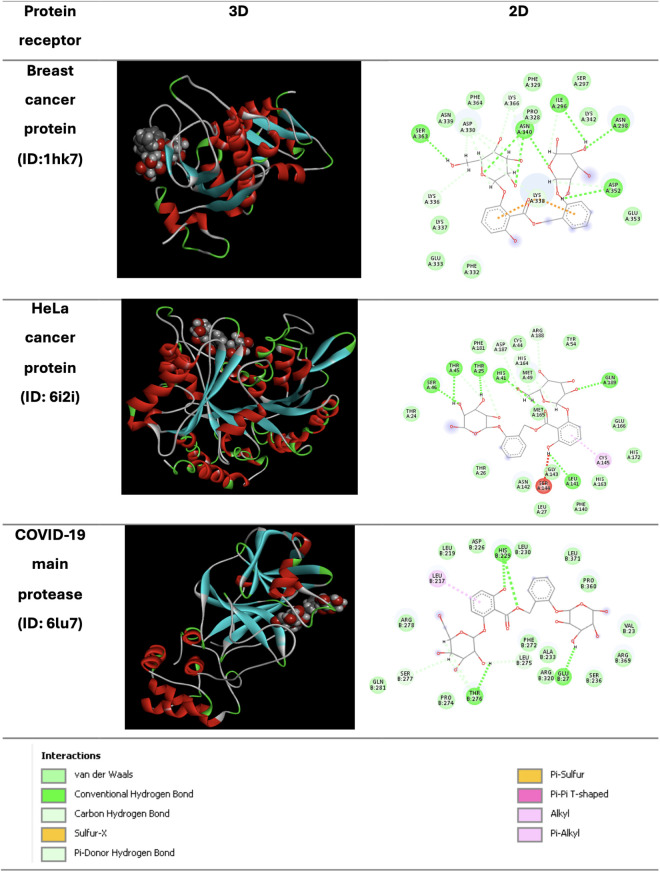
2D and 3D interaction profiles of the henryoside ligand with breast cancer protein (PDB ID: 1HK7), HeLa cancer protein (PDB ID: 6I2I), and SARS-CoV-2 main protease (PDB ID: 6LU7).

The docking scores representing the binding affinities of the explored compounds to cancer-related proteins and the SARS-CoV-2 main protease are presented in Table 6 (E. foeminea fruit extract) and Table 7 (E. foeminea branch extract), with scores ranging from −4.4 to −8.5 kcal/mol. Overall, the compounds identified in the fruit extract of E. foeminea showed stronger binding interactions than those from the branch extract. Among all the tested compounds, henryoside exhibited the highest docking free energy values across all target receptors. Conversely, guajavarin, neohancoside, and piperine showed comparatively lower binding scores. The docking structures for henryoside are presented as 2D and 3D images in Fig 10. Interestingly, the ligand henryoside interacted with key amino acid residues in the breast cancer protein (1HK7), such as asparagine (ASP-352), asparagine (ASN-340), and serine (SER-363), primarily through van der Waals forces and hydrogen bonding. These interactions were also observed between henryoside and the HeLa protein, where functional amino residues such as histidine (HIS-43), threonine (THR-45), and leucine (LEU-189). Additionally, pi-alkyl binding with cysteine (CYS-145) was observed, facilitated by a phenyl ring in the ligand. The optimal pose for henryoside interaction against the SARS-CoV-2 protein exhibited van der Waal forces, hydrogen bonding, and pi-alkyl binding with threonine (THR-276), serine (SER-277), histidine (HIS-229), glutamine (GLU-27), and leucine (LEU-217).

**Table 6:** Free energy binding scores (kcal/mol) of E. foeminea fruit compounds docked against breast cancer protein (PDB ID: 1HK7), HeLa protein (PDB ID: 6I2I), and the SARS-CoV-2 main protease (PDB ID: 6LU7).

No.	compound	Protein/score		
		Breast Protein (1HK7)(Kcal/mol)	Hela Protein (6I2I)(Kcal/mol)	SARS-CoV-2 Main Protease (6LU7)(Kcal/mol)
1	Epicatchin	−5.1858263	−5.21540356	−5.82963514
2	Djalonensone	−5.1899910	−5.14765978	−5.85130882
3	Cis-melilotoside	−6.3885827	−5.63430834	−6.69275236
4	Tigonelline	−4.5745635	−4.49371099	−4.61012983
5	Norharmane	−4.3828272	−4.53674746	−4.68390083
6	Neohancoside	−6.61995125	−6.91260672	−7.48542166
7	Guajavarin	−6.80856371	−6.26820993	−8.17760658
8	Henryoside	−7.63489246	−7.88632917	−8.51492786
9	Kaempferol	−5.36890364	−5.12310028	−5.92378044
10	isorhamnetin	−6.29753351	−6.26473665	−7.88034725

**Table 7:** Free energy binding scores (kcal/mol) of *E. foeminea* branch compounds docked against the breast cancer protein (PDB ID: 1HK7), HeLa protein (PDB ID: 6I2I), and the SARS-CoV-2 main protease (PDB ID: 6LU7).

No.	Compound		Protein/Score	
		Breast Cancer Protein (1HK7)	HeLa Protein (6I2I)	SARS-CoV-2 main protease (6LU7)
1	6-Methylquinoline	−5.21540356	−4.3966403	−4.65645885
2	Piperine	−7.14966202	−5.64066648	−6.76600838
3	Galdosol	−5.54618788	−5.29431057	−6.58433914

## Discussion

This study offers new insights into the chemical composition and biological activities of the hot aqueous extract of E. foeminea, a medicinal plant that has been largely underexplored in the scientific literature. The primary objective of this study was to examine the chemical profile of the plant using a metabolomics-based approach that combines NMR, GC − MS, and LC − MS techniques. Through this approach, we identified diverse secondary metabolites, many of which had not been reported prior to this study. A secondary objective was to assess the bioactivity of the secondary metabolites present in the hot aqueous extracts of the fruits and branches of the plant. By addressing these aims, our study seeks to enhance the understanding of the traditional applications and therapeutic potential of the plant.

The liquid-state NMR spectra revealed a chemical fingerprint of the plant extracts, indicating a predominance of polysaccharides. These findings were corroborated by the solid-state NMR, which also showed higher concentrations of phenols, flavonoids, and substituted aromatics, indicating that the hot aqueous solvent may not effectively solubilize all the potential bioactive compounds present in the plant. Further analysis using GC − MS and UHPLC−MS in positive and negative modes was conducted to provide a more comprehensive metabolite profiling of E. foeminea. This analysis confirmed the NMR findings, revealing a high concentration of polysaccharides, while also identifying other compounds, such as phenols, flavonoids, fatty acids, and organic acids. E. foeminea is well known for its alkaloid compound content [79] particularly in the leaves, some of which were also detected in our study. Notably, trigonelline, known for its hypoglycemic, neuroprotective, and antibacterial activity [80] may be of particular interest. Norharmane, a β-carboline alkaloid, has shown anti-influenza activity [81] and is also known for its neuroprotective, antioxidant, and anti-inflammatory properties [82]. To the best of our knowledge, norharmane has not previously been identified in E. foeminea. The plant is also recognized for its flavonoid content [17,83]. In this study, we identified epicatechin, kaempferol, guajavarin, and isorhamnetin-3-O-glucoside, among others. Phenolic compounds, including flavonoids and tannins, exhibit a wide range of biological activities. These compounds can modulate signaling pathways, induce apoptosis, and inhibit tumor cell growth [84]. Therefore, isolating these compounds from E. foeminea could support the traditional use of the plant in cancer treatment. Other noteworthy aromatic compounds identified in the aqueous extracts include vanillic acid and gallic acid, which are well known for their antioxidant, anti-inflammatory, and anticancer activities [85]. Pantothenic acid (vitamin B_5_)_,_ recognized for its antioxidant activity, was also detected using MS [86,87]. Furthermore, several fatty acids were also detected, some of which were identified for the first time in the hot aqueous extract of E. foeminea. Among these were 2-hydroxypalmitic acid [88] and azelaic acid (also known as nonanedioic acid), both of which are known for their anti-inflammatory effects [89]. Galdosol, a terpenoid recognized for its potent antioxidant activity, was also detected for the first time in this plant species [90].

The antioxidant capacity of E. foeminea was supported by a high TPC, particularly in the branch extract, which also showed superior DPPH radical scavenging activity. The higher bioactivity observed in the branch extract may be attributed to limitations in the extraction method, which failed to extract known antioxidant unsaturated fatty acids, flavonoids, and phenols from the fruits. However, this result may also reflect a lower correlation between the antioxidant compounds detected and the DPPH and TPC assays. Flavonoids interact with one another, and these interactions can be either synergistic or antagonistic, depending on their ratios and other factors, leading to varying antioxidant capabilities and, consequently, different bioactivity outcomes [91–93].

Regarding the anticancer activity, the MTT assay indicated that E. foeminea aqueous extract may exhibit anticancer activities and be a promising basis for anticancer drug development, particularly for breast cancer. Our study showed moderate cytotoxic activity in the branch and fruit extracts on the human breast cancer cell line (MCF-7), indicating the valid traditional use of the plant in hot aqueous infusions as an anticancer therapy [94]. Previous studies have also shown the anticancer potential of E. foeminea [17,23,94,95]. although in those studies, extractions were performed using organic solvents rather than water. Our findings highlight the significance of aqueous extracts, which more closely align with traditional preparation methods, and suggest that water-based extractions may offer a viable and accessible alternative. The results of this study indicate that the cytotoxicity of E. foeminea fruit extracts against cancer cells is greater than that of green branches. This enhanced effectiveness is likely attributed to a higher concentration of anticancer compounds in the fleshy fruits. It is well established that the biosynthesis of the plant occurs in its green tissues, which subsequently transform these essential compounds into storage organs, such as the fruits of E. foeminea. A literature review conducted to identify phytoconstituents that may have contributed to the observed anticancer effects revealed that several molecules identified through GC − MS and LC − MS analyses possess anticancer properties. Notable among these are trigonelline [61,96], norharmane [63,97], epicatechin [98,99], and kaempferol [100] found in the fruit components of E. foeminea as well as piperine [101,102], and azelaic acid [77,103] which were detected in the branches.

To gain a more detailed understanding of the biological potential of E. foeminea, molecular docking was performed to predict the interactions between selected ligands and three target proteins: the breast cancer protein (PDB ID: 1HK7), the HeLa cancer protein (PDB ID: 6I2I), and the SARS-CoV-2 main protease (PDB ID: 6LU7). Among the tested compounds, henryoside showed the strongest binding affinities, with scores of −7.6, −7.9, and −8.5 kcal/mol against the breast cancer protein, HeLa protein, and SARS-CoV-2 main protease, respectively [46,47]. Henryoside, an acylated salicin bis-glucoside originally isolated from Viburnum veitchii, was first identified in the fruit extract. This compound is known for its spasmolytic and uterotonic properties [104], and contains functional groups that may interact with various signaling pathways involved in different stages of cell transformation [105]. Several studies have suggested that these functional groups can act as anticancer inhibitors by binding to active sites on cancer cells, thereby inducing apoptosis, autophagy, and cell cycle arrest with high specificity [106,107]. Henryoside remains an underexplored plant metabolite, with limited data on its chemical properties, biological activities, and potential therapeutic applications [108]. Therefore it warrants further research.

Furthermore, polyphenol compounds have also shown antiviral activities [109]. In our molecular docking analysis, the flavanol molecule, guajavarin, ranked second in terms of free binding energy affinity to the selected proteins. Guajavarin is a derivative of quercetin, which is well known as an effective cancer prevention agent [110]. Additionally, neohancoside, an alkaloid molecule, showed good binding affinities to the target proteins, as shown in Table 6. These three bioactive molecules—guajavarin, henryoside, and neohancoside—among other constituents identified in E. foeminea fruits, showed promising interactions that may explain the in vitro anticancer bioassay results.

The identification of compounds in this study is based on untargeted metabolomic profiling, which relies on spectral matching and comparison with reference libraries. Future research involving compound isolation and targeted analyses is required for precise validation.

## Conclusions

This study highlights the potential of the aqueous extract of the medicinal plant E. foeminea as a promising source of bioactive secondary metabolites with significant antioxidant and anticancer properties. Antioxidant assays revealed that branch extracts exhibited superior antioxidant activity, likely due to their higher TPC. Conversely, fruit extracts showed greater cytotoxic activity against MCF-7 breast cancer cells. Molecular docking analysis identified specific compounds—henryoside, guajavarin, and neohancoside—as promising anticancer and antiviral agents. These findings underscore the therapeutic potential of E. foeminea and its potential as a source for the development of novel bioactive compounds. Comprehensive metabolite profiling conducted using NMR, GC − MS, and LC − MS analyses provides a strong foundation for understanding the chemical diversity of this plant and supports further exploration of its wide range of bioactive compounds. Untargeted metabolomic analysis revealed distinct chemical profiles in the branch and fruit extracts, with 232 metabolites identified in the fruit samples and 117 in the branch samples. The antioxidant activity observed in E. foeminea indicates its potential to protect against diseases associated with oxidative stress and to contribute to cancer prevention. These findings emphasize the relevance of this plant in the development of natural therapeutic agents. Overall, the results of this study support the traditional use of E. foeminea, highlighting the relevance of ancestral practices and the importance of re-examining natural products as a foundation for sustainable pharmaceutical development. Future research should focus on the isolation and characterization of specific bioactive compounds to fully explore the medicinal potential of the aqueous extract of E. foeminea and to facilitate the development of novel natural therapeutics.

## Supporting information

S1 File Supporting Information for Ephedra foeminea extract analyses. This file includes: S1 Table. Molecular docking selected compounds; S2 Table. Compounds detected by UHPLC-MS(timsTOF) in E.foeminea branches extract; S3 Table. Compounds detected by GC-MS in E.foeminea branches extracts; S4 Table. Compounds detected by UHPLC-MS(timsTOF) in E.foeminea fruit extract; S5 Table. Compounds detected by GC-MS in E.foeminea fruit extract; S1 Fig. Screenshot of Chenomx Profiler (Chenomx Suite 9.0, Alberta, Canada) software; S2 Fig. Screenshot of Chenomx Profiler (Chenomx Suite 9.0, Alberta, Canada) software.; S3 Fig. Screenshot of Chenomx Profiler (Chenomx Suite 9.0, Alberta, Canada) software window; S4 Fig. Screenshot of Chenomx Profiler (Chenomx Suite 9.0, Alberta, Canada) software window.;S5 Fig. Screenshot of Chenomx Profiler (Chenomx Suite 9.0, Alberta, Canada) software window; S6 Fig. Screenshot of Chenomx Profiler (Chenomx Suite 9.0, Alberta, Canada) software window;S6 Table: Tentative NMR assignments of selected metabolites detected in Ephedra foeminea extract; S7 Fig. Full NMR spectra of E. foeminea.
(DOCX)
