# Supplementary material for: Medicinal effects of Ephedra foeminea aqueous extracts: Metabolomic characterization, biological evaluation, and molecular docking
Source: PLoS One. 2025 Jul 24;20(7):e0328995. doi: 10.1371/journal.pone.0328995 (PMC12289038; doi:10.1371/journal.pone.0328995)
Supplement: S1 File — This file includes: S1 Table. Molecular docking selected compounds; S2 Table. Compounds detected by UHPLC-MS(timsTOF) in E.foeminea branches extract; S3 Table. Compounds detected by GC-MS in E.foeminea branches extracts; S4 Table. Compounds detected by UHPLC-MS(timsTOF) in E.foeminea fruit extract; S5 Table. Compounds detected by GC-MS in E.foeminea fruit extract; S1 Fig. Screenshot of Chenomx Profiler (Chenomx Suite 9.0, Alberta, Canada) software; S2 Fig. Screenshot of Chenomx Profiler (Chenomx Suite 9.0, Alberta, Canada) software.; S3 Fig. Screenshot of Chenomx Profiler (Chenomx Suite 9.0, Alberta, Canada) software window; S4 Fig. Screenshot of Chenomx Profiler (Chenomx Suite 9.0, Alberta, Canada) software window.;S5 Fig. Screenshot of Chenomx Profiler (Chenomx Suite 9.0, Alberta, Canada) software window; S6 Fig. Screenshot of Chenomx Profiler (Chenomx Suite 9.0, Alberta, Canada) software window;S6 Table: Tentative NMR assignments of selected metabolites detected in Ephedra foeminea extract; S7 Fig. Full NMR spectra of E. foeminea. (DOCX) [file pone.0328995.s001.docx]

Medicinal Effects of *Ephedra foeminea* Aqueous Extracts: Metabolomic Characterization, Biological Evaluation, and Molecular Docking

**Giulia Bennici^1^, Inas Al Younis^1^, Abeer Sharfalddin^2^, Mutaz Akkawi^3^, Fuad Al-Rimawi^4^, Khaled Sawalha^3^, Abdul-Hamid Emwas^5*^, Mariusz Jaremko^1*^.**

^1^Division of Biological and Environmental Sciences and Engineering (BESE), King Abdullah University of Science and Technology (KAUST), Thuwal, Saudi Arabia

^2^Department of Chemistry, Faculty of Science, King Abdul Aziz University, Jeddah

^3^Biology Department, Faculty of Science and Technology, Al-Quds University, Jerusalem, Palestine

^4^Chemistry Department, Faculty of Science and Technology, Al-Quds University, Jerusalem, Palestine

^5^Core Lab of NMR, King Abdullah University of Science and Technology (KAUST), Thuwal, Saudi Arabia

***** Correspondence: [abdelhamid.emwas@kaust.edu.sa](mailto:abdelhamid.emwas@kaust.edu.sa); [mariusz.jaremko@kaust.edu.sa](mailto:mariusz.jaremko@kaust.edu.sa).

**S1 Table**. **Molecular docking selected compounds.**

| A)Branches | No. | Compound name | Structure | References |
| --- | --- | --- | --- | --- |
|  | 1 | Trigonelline | 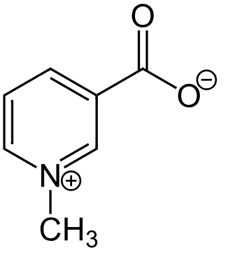 | [1], [2] |
|  | 2 | Norharmane | 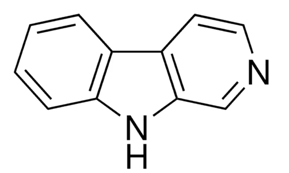 | [3], [4] |
|  | 3 | Epicatechin | 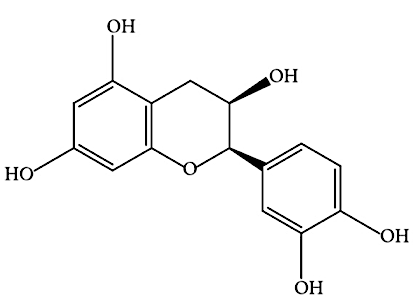 | [5], [6] |
|  | 4 | isorhamnetin-3-O-glucoside | 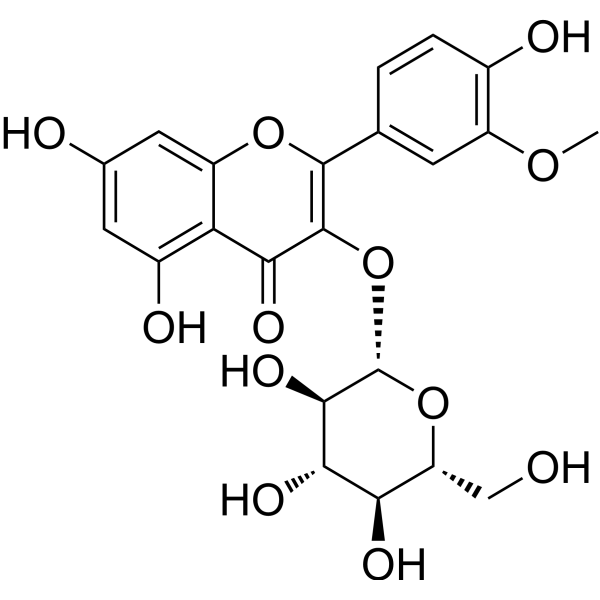 | [7], [8], [9] |
|  | 5 | Djalonensone | 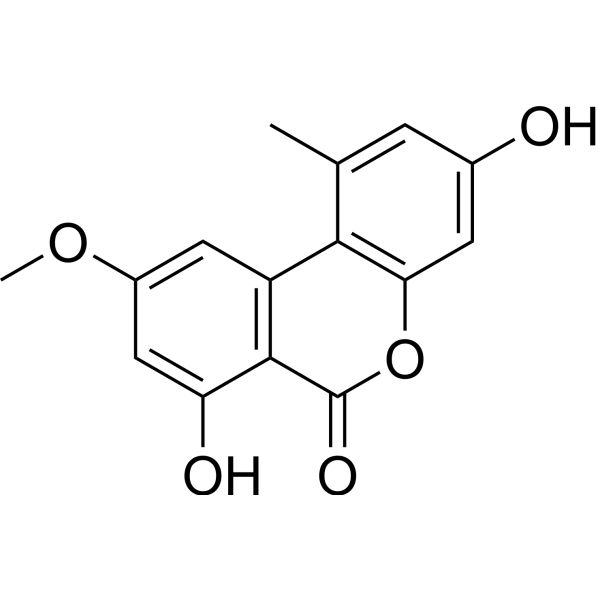 | [10] |
|  | 6 | Kaempferol | 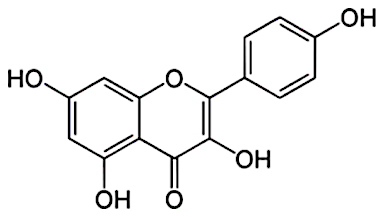 | [11], [12], [13] |
|  | 7 | Cis-Melilotoside | 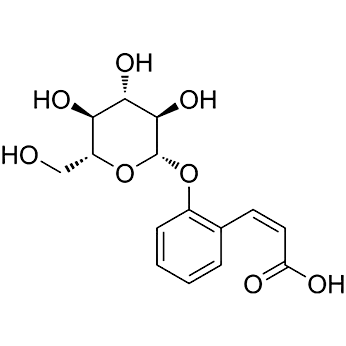 | [14], [15] |
|  | 8 | Henryoside | 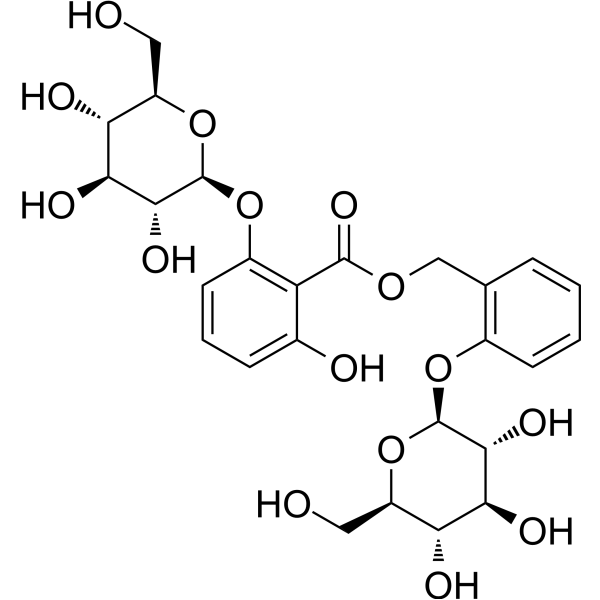 | [16] |
|  | 9 | Neohancoside D | 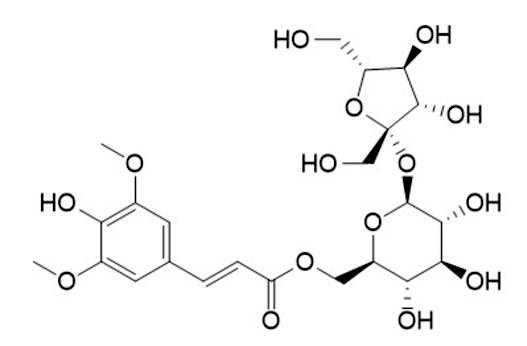 | [17], [18] |
|  | 10 | Guajavarin | 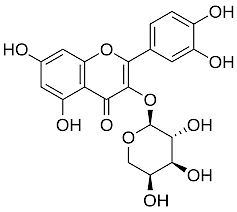 | [19], [20] |
| B)Fruits | 11 | 6-Methylquinoline | 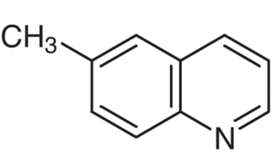 | [21], [22] |
|  | 12 | Piperine | 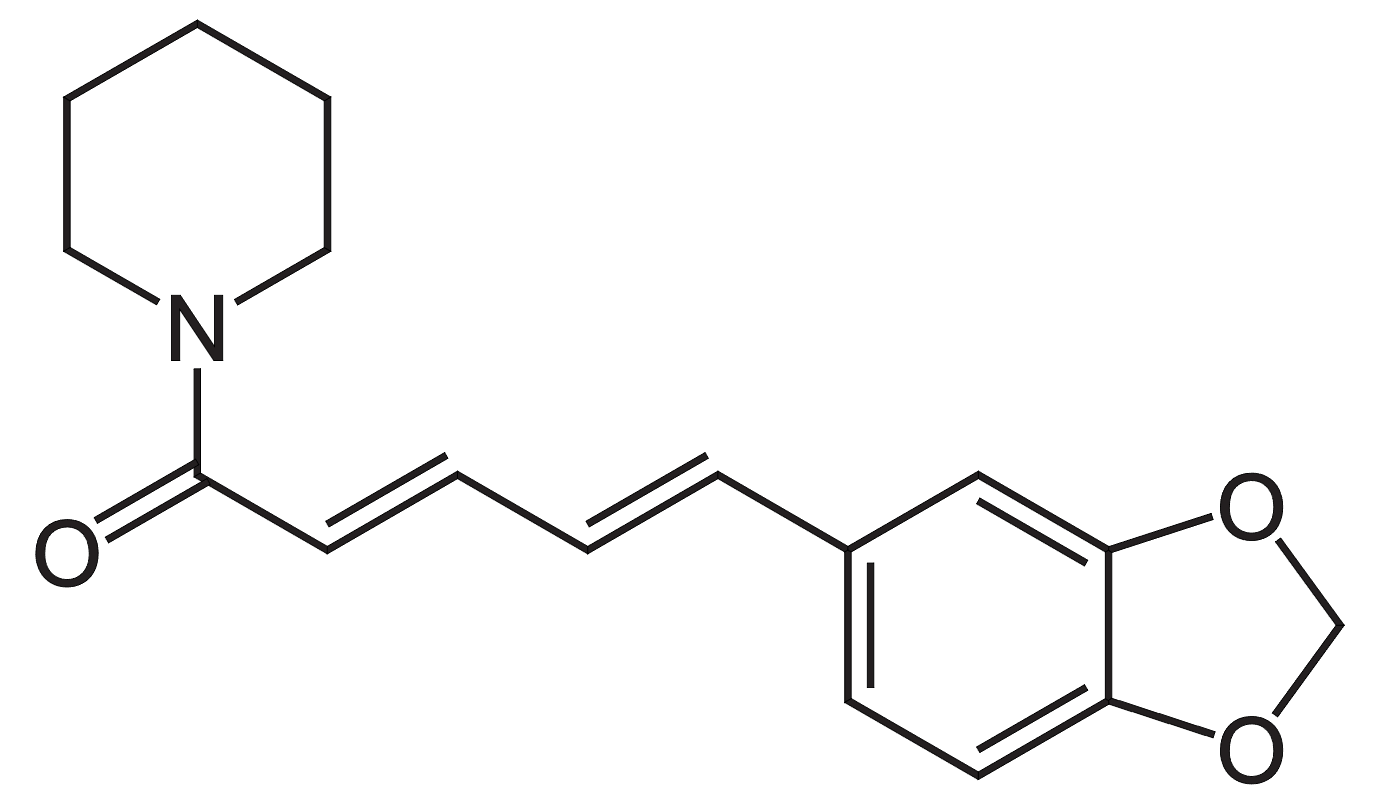 | [23], [24] |
|  | 13 | Galdosol | 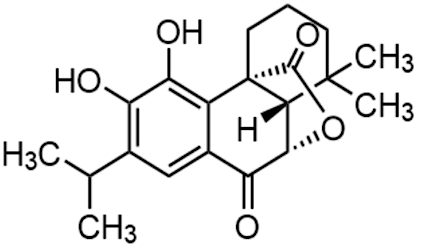 | [25], [26], [27], [28] |

Structure of the selected compounds for molecular docking analysis from *Ephedra foeminea*. Section A) Branches and section B) Fruits.

**S2 Table. Compounds detected by UHPLC-MS(timsTOF) in *E.foeminea* branches extract.**

| Name | Molecular Formula | MS/MS score | Ions | m/z meas. | RT [min] |
| --- | --- | --- | --- | --- | --- |
| Tri(3-chloropropyl) phosphate | C9H18Cl3O4P | | [M+H]+, [M+Na]+ | 327.0082 | 11.82 |
| N,N-Dimethyldodecan-1-amine | C14H31N |  | [M+H]+ | 214.25314 | 10.25 |
| N,N-Dimethyldodecan-1-amine | C14H31N |  | [M+H]+ | 214.25277 | 10.43 |
| D-erythro-Sphingosine C-17 | C17H35NO2 |  | [M+H]+ | 286.27438 | 10.38 |
| Cis-2-Decenoic acid | C10H18O2 |  | [M+H]+ | 171.13813 | 11.14 |
| Adenosine | C10H13N5O4 | | [M+H]+ | 268.10415 | 0.94 |
| 5,8,11-Eicosatriynoic Acid | C20H28O2 |  | [M+H]+ | 301.21616 | 13.51 |
| 10E,12Z-Octadecadienoic acid | C18H32O2 |  | [M+H]+ | 281.24775 | 13.2 |
| 1-Hexadecyl-sn-glycero-3-phosphocholine | C24H52NO6P | | [M+H]+, [M+Na]+ | 482.36061 | 14.04 |
| N-(1-Phenylethyl)cyclohexanecarboxamide | C15H21NO | 943.1 | [M+H]+ | 232.16974 | 11.05 |
| Tris(butoxyethyl)phosphate | C18H39O7P | 979.5 | [M+H]+, [M+Na]+, [M+H]+ | 399.25092 | 13.88 |
| 4-Methyl-N-(4-methylphenyl)benzamide | C15H15NO | 912.2 | [M+H]+ | 226.12301 | 11.42 |
| 2-Methylbutyroylcarnitine | C12H23NO4 | 987.2 | [M+H]+ | 246.1702 | 4.24 |
| 6-Oxocativic acid | C20H32O3 | 952.5 | [M+H]+ | 321.24216 | 11.93 |
| Kahweol | C20H26O3 | 662.8 | [M+H]+ | 315.19547 | 11.53 |
| TOLPERISONE | C16H23NO | 644.5 | [M+H]+ | 246.18522 | 12.05 |
| Dibutyl sebacate | C18H34O4 | 777.5 | [M+H]+ | 315.25304 | 16.33 |
| Dodemorph | C18H35NO | 665.5 | [M+H]+ | 282.2792 | 16.44 |
| 3-ketosphingosine | C18H35NO2 | 644.8 | [M+H]+ | 298.27419 | 14.28 |
| 19-Norepiandrosterone | C18H28O2 | 705 | [M+H]+ | 277.21635 | 11.95 |
| 19-Norepiandrosterone | C18H28O2 | 715.5 | [M+H]+ | 277.21635 | 13.37 |
| (4S,4AR)-4-(Hydroxymethyl)-3,4a,8,8-tetramethyl-4a,5,6,7,8,8a-hexahydronaphthalen-1(4H)-one | C15H24O2 | 634.7 | [M+H]+ | 237.18513 | 11.62 |
| Heptapropylene glycol | C21H44O8 | 852.7 | [M+H]+, [M+Na]+ | 425.31119 | 9.75 |
| R-Palmitoyl-(2-methyl) Ethanolamide | C19H39NO2 | 849.6 | [M+H]+ | 314.30566 | 11.38 |
| 19-Norepiandrosterone | C18H28O2 | 694 | [M+H]+ | 277.21654 | 12.74 |
| 3-(4-Toluidino)-2-cyclohexen-1-one | C13H15NO | 655.8 | [M+H]+ | 202.12302 | 6.72 |
| LPC 20:4 | C28H50NO7P | 458.4 | [M+H]+ | 544.34032 | 13.23 |
| Triphenyl phosphate | C18H15O4P | 822.5 | [M+H]+ | 327.07811 | 13.4 |
| 3-Cyclohexyl-1,1-dimethylurea | C9H18N2O | 883.2 | [M+H]+ | 171.14913 | 8.56 |
| Piperine | C17H19NO3 | 896.8 | [M+H]+ | 286.14387 | 11.8 |
| Nootkatone | C15H22O | 750 | [M+H]+ | 219.17445 | 13.44 |
| 2-Phenyl-1-(piperidin-1-yl)butan-1-one | C15H21NO | 749.3 | [M+H]+ | 232.16971 | 11.56 |
| N-Tert-Butyl-.alpha.-phenylnitrone | C11H15NO | 885.5 | [M+H]+ | 178.1228 | 11.1 |
| 6-Methylthioguanine | C6H7N5S | 891.7 | [M+H]+ | 182.04966 | 4.7 |
| 4-(1-Phenylcyclohexyl)morpholine | C16H23NO | 668.7 | [M+H]+ | 246.18542 | 12.13 |
| 2-Phenyl-1-(piperidin-1-yl)butan-1-one | C15H21NO | 819.8 | [M+H]+ | 232.16978 | 11.63 |
| N-Oleoylglycine | C20H37NO3 | 723.8 | [M+H]+ | 340.28484 | 15.66 |
| 2-Methyl-N-(4-Methylphenyl)alanine | C11H15NO2 | 674.9 | [M+H]+ | 194.11781 | 20.32 |
| Triphenylphosphine oxide | C18H15OP | 705.8 | [M+H]+ | 279.09359 | 10.85 |
| 15d-PGA1 | C20H30O3 | 754.9 | [M+H]+ | 319.22642 | 13.37 |
| Linoleic acid | C18H32O2 | 764.1 | [M+H]+ | 281.24791 | 14.14 |
| 9-OxoODE | C18H30O3 | 845.4 | [M+H]+ | 295.22733 | 14.46 |
| 9-Benzyl-9-azabicyclo[3.3.1]nonan-3-one | C15H19NO | 999.7 | [M+H]+ | 230.154 | 11.41 |
| Palmitic amide | C16H33NO | 901.1 | [M+H]+ | 256.26358 | 16.25 |
| 1-(2-Hydroxyethyl)-2,2,6,6-tetramethyl-4-piperidinol | C11H23NO2 | 940.7 | [M+H]+ | 202.18048 | 0.63 |
| Linoleic acid | C18H32O2 | 919.9 | [M+H]+ | 281.24751 | 15.65 |
| Tributyl phosphate | C12H27O4P | 999.7 | [M+H]+ | 267.17196 | 13.34 |
| Palmitoylcarnitine | C23H45NO4 | 987.6 | [M+H]+ | 400.34225 | 12.42 |
| PARAXANTHINE | C7H8N4O2 | 961.4 | [M+H]+ | 181.07213 | 5.89 |
| 5-Hydroxy-6E,8Z,11Z,14Z-eicosatetraenoic acid, 1,5-lactone | C20H30O2 | 927.8 | [M+H]+ | 303.232 | 14.26 |
| Iodotyrosine | C9H10INO3 | 960.7 | [M+H]+ | 307.97798 | 5.19 |
| Caffeine | C8H10N4O2 | 974.7 | [M+H]+ | 195.08782 | 6.57 |
| Di-N-octyl phthalate | C24H38O4 | 990 | [M+Na]+, [M+H]+, [M+H]+ | 413.26651 | 18.03 |
| Carbamazepine | C15H12N2O | 977.4 | [M+H]+ | 237.10255 | 9.71 |
| Vidarabine | C10H13N5O4 | 995.9 | [M+H]+ | 268.10442 | 0.69 |
| LPC O-16:1 | C24H50NO6P | 931.9 | [M+H]+ | 480.34528 | 13.06 |
| LPC O-18:0 | C26H56NO6P | 940 | [M+H]+ | 510.39255 | 15.51 |
| 1H-Indazole-4-carboxamide, N-[(1,2-dihydro-6-methyl-2-oxo-4-propyl-3-pyridinyl)methyl]-1-(1-methylethyl)-6-[2-(4-methyl-1-piperazinyl)-4-pyridinyl]- | C31H39N7O2 | 908.5 | [M+H]+, [M+Na]+ | 542.32287 | 12.8 |
| PC 9:0_16:0 | C33H66NO8P | 951.1 | [M+H]+ | 636.46 | 16.18 |
| diethyltoluamide | C12H17NO | 956.4 | [M+H]+, [M+H]+, [M+Na]+ | 192.13887 | 10.43 |
| (-)-trans-C75 | C14H22O4 | 934.7 | [M-H]- | 253.14467 | 11.16 |
| 1,11-Undecanedicarboxylic acid | C13H24O4 | 849.7 | [M-H]-, [M-H-H2O]- | 243.16051 | 11.48 |
| 11-Hydroxyundecanoic acid | C11H22O3 | 966 | [M-H]- | 201.1499 | 12.53 |
| 12(13)-Epoxy-9Z-octadecenoic acid | C18H32O3 | 962.7 | [M-H]- | 295.22811 | 13.91 |
| 2-Benzoylbenzoic acid | C14H10O3 | 981 | [M-H]- | 225.0559 | 10.47 |
| 2-Decyl-3-hydroxypentanedioic acid | C15H28O5 | 972.7 | [M-H]- | 287.18641 | 11.32 |
| 2-Hydroxymyristic Acid | C14H28O3 | 999.7 | [M-H]- | 243.19662 | 13.39 |
| 2-Hydroxypalmitic acid | C16H32O3 | 999.7 | [M-H]- | 271.22782 | 14.26 |
| 2-Iodoisophthalic acid | C8H5IO4 | 999.7 | [M-H]- | 290.91602 | 13.12 |
| 2-Phenyl-1H-benzimidazole-5-sulfonic acid | C13H10N2O3S | 995.5 | [M-H]- | 273.03437 | 6.83 |
| 3-(2,4-Dimethoxyanilino)-3-oxopropanoic acid | C11H13NO5 | 997.1 | [M-H]- | 238.07217 | 18.94 |
| 3-Bromo-5-iodobenzoic acid | C7H4BrIO2 | 998.8 | [M-H]- | 324.83679 | 11.34 |
| 3,5-Di-tert-butyl-4-hydroxybenzoic acid | C15H22O3 | 980.7 | [M-H]- | 249.14998 | 12.78 |
| 3,5-Diiodobenzoic acid | C7H4I2O2 | 999.7 | [M-H]- | 372.82278 | 11.81 |
| 4-(Butoxycarbonyl)benzoic acid | C12H14O4 | 997.6 | [M-H]- | 221.08186 | 10.81 |
| 4-formyl Indole | C9H7NO | 922 | [M-H]- | 144.0456 | 8.28 |
| 5,6-Dihydroxy-8Z,11Z,14Z-eicosatrienoic acid | C20H34O4 | 939.4 | [M-H]- | 337.23806 | 11.97 |
| 9-(2,3-Dihydroxypropoxy)-9-oxononanoic acid | C12H22O6 | 982.2 | [M-H]- | 261.13452 | 8.12 |
| Dodecylbenzenesulfonic acid | C18H30O3S | 991.5 | [M-H]- | 325.18429 | 13.27 |
| Galdosol | C20H24O5 | 999.9 | [M-H]- | 343.15496 | 11.03 |
| Hexadecanedioic acid | C16H30O4 | 973.3 | [M-H]-, [M-H-H2O]- | 285.20724 | 13.54 |
| Ile-Phe | C15H22N2O3 | 937.7 | [M-H]- | 277.15581 | 6.42 |
| L-TRYPTOPHAN | C11H12N2O2 | 993.3 | [M-H]- | 203.08262 | 4.78 |
| Mono-2-ethylhexyl phthalate | C16H22O4 | 920.8 | [M-H]- | 277.145 | 13.76 |
| Nonanedioic acid | C9H16O4 | 975.5 | [M-H]- | 187.09763 | 8.52 |
| Octadecanedioic acid | C18H34O4 | 862.5 | [M-H]- | 313.23845 | 14.96 |
| Phosphonic acid, P-[(3R)-3-amino-4-[(3-hexylphenyl)amino]-4-oxobutyl]- | C16H27N2O4P | 971.3 | [M-H]- | 341.16372 | 10.05 |
| Tetradecanedioic acid | C14H26O4 | 989.4 | [M-H]- | 257.17604 | 12.16 |
| Undecanedioic acid | C11H20O4 | 977.5 | [M-H]- | 215.12888 | 10.06 |

**S3 Table. Compounds detected by GC-MS in *E.foeminea* branches extract.**

| Name | RT [min] | Delta RI |
| --- | --- | --- |
| 2-Hydroxybutanedioic acid, 3TMS | 11.689 | 45 |
| 4-Hydroxybenzaldehyde, 1MOX, 1TMS | 12.611 | 46 |
| Arabinose, 1MOX, 4TMS | 14.596 | 5 |
| Azelaic acid, 2TMS | 16.909 | 45 |
| Caprylic acid, 1TMS | 8.885 | 30 |
| D-(+)-Xylose, 1MOX, 4TMS | 15.331 | 33 |
| Gallic acid, 4TMS | 18.955 | 30 |
| Glycerol, 3TMS | 9.066 | 15 |
| Malic acid, 3TMS | 7.027 | - |
| Melibiose isomer 1, 8TMS | 26.135 | 42 |
| meso-Erythritol, 4TMS | 12.874 | 32 |
| Rhamnose isomer 1, 1MOX, 4TMS | 15.858 | 9 |
| Rhamnose isomer 1, 4TMS | 14.613 | 13 |
| Ribose isomer 1, 4TMS | 14.174 | 21 |
| Ribose isomer 2, 1MOX, 4TMS | 15.022 | 24 |
| Succinic acid, 2TMS | 9.724 | 5 |
| Sucrose, 8TMS | 26.197 | 7 |
| Thymidine, 2TMS | 23.171 | 16 |
| Uridine, 3TMS | 23.484 | 6 |

**S4 Table**. **Compounds detected by UHPLC-MS(timsTOF) in *E.foeminea* fruit extract.**

| Name | Molecular Formula | MS/MS score | Ions | m/z meas. | RT [min] |
| --- | --- | --- | --- | --- | --- |
| (+/-)8,9-DiHETrE | C20H34O4 | 1000 | [M-H]- | 337.23851 | 12.62 |
| (±)13-HpODE | C18H32O4 |  | [M-H]- | 311.22294 | 11.69 |
| (7Z)-14-hydroxy-10,13-dioxoheptadec-7-enoic acid | C17H28O5 | 976.1 | [M-H]- | 311.18656 | 11.46 |
| (dimethylcarbamoyl)methyl 3-methoxybenzoate | C12H15NO4 | 1000 | [M-H]- | 236.09302 | 6.16 |
| (methylcarbamoyl)methyl 2-{[1,1'-biphenyl]-4-yl}acetate | C17H17NO3 | 950.3 | [M-H]- | 282.11368 | 10.32 |
| [3-(methoxycarbonyl)furan-2-yl]methyl 7-methyl-[1,2,4]triazolo[1,5-a]pyrimidine-2-carboxylate | C14H12N4O5 | 937.8 | [M-H]- | 315.07228 | 6.74 |
| 1-[1-(4-fluorophenyl)pyrazolo[3,4-d]pyrimidin-4-yl]-4-(thiophen-3-ylmethyl)piperazine | C20H19FN6S | 911.8 | [M-H]- | 393.13031 | 6.95 |
| 1-[2-(3,4-dimethoxyphenyl)-2-oxoethyl]-5-(trifluoromethyl)pyridin-2-one | C16H14F3NO4 | 1000 | [M-H]- | 340.07989 | 9.23 |
| 1-{[(6-acetyl-2H-1,3-benzodioxol-5-yl)carbamoyl]methyl}cyclohexane-1-carboxylic acid | C18H21NO6 | 991.3 | [M-H]- | 346.12973 | 6.36 |
| 1-O-(2R-methoxy-4Z-tetradecenyl)-sn-glycerol | C18H36O4 | 919 | [M-H]- | 315.25427 | 12.99 |
| 1,3-dimethyl-7-(2-oxo-2-phenylethyl)purine-2,6-dione | C15H14N4O3 | 1000 | [M-H]- | 297.09839 | 6.53 |
| 1'-[(5-methyl-1,3,4-oxadiazol-2-yl)methyl]-2,3-dihydrospiro[1-benzopyran-4,4'-imidazolidine]-2',5'-dione | C15H14N4O4 | 1000 | [M-H]- | 313.09279 | 6 |
| 11S-HETE | C20H32O3 | 1000 | [M-H]- | 319.22791 | 13.86 |
| 13-(2-methyl-1,3-thiazol-4-yl)-1,8,10,12-tetraazatricyclo[7.4.0.0^{2,7}]trideca-2,4,6,8,10-pentaen-11-amine | C13H12N6S | 1000 | [M-H]- | 283.07607 | 5.44 |
| 13-(naphthalen-2-yl)-1,8,10,12-tetraazatricyclo[7.4.0.0^{2,7}]trideca-2,4,6,8,10-pentaen-11-amine | C19H15N5 | 1000 | [M-H]- | 312.12424 | 9.48 |
| 15S-Hydroperoxy-11Z,13E-eicosadienoic acid | C20H36O4 | 778.4 | [M-H]- | 339.25443 | 13.64 |
| 2-(1-benzofuran-2-ylformamido)-N-methylacetamide | C12H12N2O3 | 946.6 | [M-H]- | 231.07766 | 4.48 |
| 2-(1-cyclopropyl-2,5-dimethylpyrrol-3-yl)-2-oxoethyl 4-hydroxybenzoate | C18H19NO4 | 1000 | [M-H]- | 312.12428 | 10.08 |
| 2-(1,3-dimethyl-2,6-dioxopurin-7-yl)-N-(2-methylpropyl)acetamide | C13H19N5O3 | 1000 | [M-H]- | 292.14024 | 1.03 |
| 2-(4-methoxyphenoxy)-N-phenylacetamide | C15H15NO3 | 980.5 | [M-H]- | 256.09832 | 9.52 |
| 2-[(4-cyclopropyl-5-methyl-1,2,4-triazol-3-yl)sulfanyl]-1-[4-(2-fluorophenyl)piperazin-1-yl]etha | C18H22FN5OS | 1000 | [M-H]- | 374.1456 | 6.61 |
| 2-[(4-methoxyphenyl)amino]benzoic acid | C14H13NO3 | 980.3 | [M-H]- | 242.08244 | 10.38 |
| 2-[ethyl({[(4-methoxyphenyl)carbamoyl]methyl})amino]-N-isopropylacetamide | C16H25N3O3 | 1000 | [M-H]- | 306.18246 | 0.64 |
| 2-isopropyl-malic acid | C7H12O5 | 1000 | [M-H]- | 175.06131 | 6.42 |
| 2-oxo-2-(2-oxopyrrolidin-1-yl)ethyl 2,4-dimethoxybenzoate | C15H17NO6 | 796.1 | [M-H]- | 306.09839 | 8.56 |
| 2,4,5-trimethoxy-N-methyl-N-[2-(3-methylphenoxy)ethyl]benzamide | C20H25NO5 | 1000 | [M-H]- | 358.16575 | 10.66 |
| 2,6Z-Nonadien-4-olide | C9H12O2 | 1000 | [M-H]- | 151.07658 | 9.08 |
| 3-(2,5-dioxopyrrolidin-1-yl)-N-phenylpropanamide | C13H14N2O3 | 1000 | [M-H]- | 245.09328 | 7.91 |
| 3-cyclopentyl-2-({[3-(2,4-dimethoxyphenyl)-1,2,4-oxadiazol-5-yl]methyl}sulfanyl)quinazolin-4-one | C24H24N4O4S | 1000 | [M-H]- | 463.14435 | 6.19 |
| 3-Methylsubericacid | C9H16O4 | 1000 | [M-H]- | 187.09777 | 8.49 |
| 3-O-alpha-L-rhamnopyranosyl-3-hydroxynonanoyl-3-hydroxydecanoic acid | C25H46O9 | 1000 | [M-H]- | 489.30677 | 14.27 |
| 3-O-Demethylamorphigenin | C22H20O7 | 925 | [M-H]- | 395.11397 | 8.95 |
| 3,12-dihydroxy palmitic acid | C16H32O4 | 1000 | [M-H]- | 287.22286 | 10.48 |
| 3,4-Dihydroxybenzoic acid | C7H6O4 | 1000 | [M-H]- | 153.01949 | 5.21 |
| 3',5'-O-Dimethylmyricetin 3-O-beta-D-2'',3''-diacetylglucopyranoside | C27H28O15 | 935.2 | [M-H]- | 591.1353 | 7.03 |
| 5-methyl-5-(4-methylphenyl)imidazolidine-2,4-dione | C11H12N2O2 | 1000 | [M-H]- | 203.08267 | 4.76 |
| 7S,10S-diHOME | C18H34O4 | 1000 | [M-H]- | 313.23848 | 12.25 |
| 9(S)-HpODE | C18H32O4 | 916.6 | [M-H]- | 311.223 | 12.51 |
| 9R-HODE | C18H32O3 | 935.3 | [M-H]- | 295.22827 | 13.86 |
| Alternariol monomethylether | C15H12O5 | 895.2 | [M-H]- | 271.06134 | 11.96 |
| Asclepin | C31H42O10 | 1000 | [M-H]- | 573.27083 | 9.1 |
| Catechin 4'-O-beta-D-glucopyranoside | C21H24O11 | 967.3 | [M-H]- | 451.1239 | 6.56 |
| Cis-Melilotoside | C15H18O8 | 995.6 | [M-H]- | 325.09325 | 6.58 |
| Colneleic acid | C18H30O3 | 1000 | [M-H]-, [M-H]-, [M-H-H2O]- | 293.21244 | 13.16 |
| Deca-4,6,8-triyne-1,1,2,3-tetraol | C10H10O4 | 948.8 | [M-H]- | 193.05071 | 8.07 |
| delta6-8-IsoF | C20H34O6 | 1000 | [M-H]- | 369.22849 | 9.32 |
| Dodecylbenzenesulfonic acid | C18H30O3S | 986.3 | [M-H]- | 325.18441 | 16.82 |
| Embelin | C17H26O4 | 909.1 | [M-H]- | 293.17623 | 12.26 |
| ethyl 2-oxo-4-(phenoxymethyl)-6-phenyl-3,6-dihydro-1H-pyrimidine-5-carboxylate | C20H20N2O4 | 1000 | [M-H]- | 351.13501 | 9.53 |
| ethyl 4-benzyl-1-ethyl-2,3-dioxoquinoxaline-6-carboxylate | C20H20N2O4 | 1000 | [M-H]- | 351.13545 | 9.92 |
| Fisetinidol-4beta-ol | C15H14O6 | 975.8 | [M-H]- | 289.07201 | 7.07 |
| Grevillic acid | C9H8O4 |  | [M-H]- | 179.03509 | 7.04 |
| Henryoside | C26H32O15 | 986.9 | [M-H]- | 583.16692 | 9.07 |
| Herbacetin 3-beta-D-glucofuranoside | C21H20O12 | 917.4 | [M-H]- | 463.08779 | 7.42 |
| Kaempferol 3-galactoside-7-rhamnoside | C27H30O15 | 1000 | [M-H]- | 593.15072 | 8.52 |
| Kaempferol 3-glucoside-7-glucuronide | C27H28O17 | 952 | [M-H]- | 623.12517 | 7.71 |
| Linoside A | C32H38O16 | 1000 | [M-H]- | 677.20842 | 8.99 |
| methyl 3-[(3-methoxyphenyl)formamido]propanoate | C12H15NO4 | 1000 | [M-H]- | 236.09309 | 6.47 |
| methyl 3-[(4-cyanophenyl)formamido]propanoate | C12H12N2O3 | 1000 | [M-H]- | 231.07752 | 6.15 |
| N-(3-oxo-2,4-dihydro-1,4-benzoxazin-6-yl)butanamide | C12H14N2O3 | 772 | [M-H]- | 233.09338 | 6.05 |
| N-(3,5-dimethoxyphenyl)-2,5-dimethylbenzamide | C17H19NO3 | 920.2 | [M-H]- | 284.1296 | 12.02 |
| N-(benzyloxy)-2-(3,7-dimethyl-2,6-dioxopurin-1-yl)acetamide | C16H17N5O4 | 1000 | [M-H]- | 342.11955 | 6.59 |
| N-[(3,5-dimethyl-1-phenylpyrazol-4-yl)methyl]-2-{[1-(3-fluorophenyl)imidazol-2-yl]sulfanyl}acetamide | C23H22FN5OS | 1000 | [M-H]- | 434.14545 | 7.37 |
| N-cyclopropyl-2-{1,3-dioxo-2-azaspiro[4.5]decan-2-yl}acetamide | C14H20N2O3 | 657.9 | [M-H]- | 263.14007 | 5.56 |
| N-ethyl-2-methyl-N-phenyl-5-(2-{[1,2,4]triazolo[4,3-a]pyridin-3-yl}pyrrolidine-1-carbonyl)benzenesulfonamide | C26H27N5O3S | 940.2 | [M-H]- | 488.17669 | 6.34 |
| N-palmitoyl phenylalanine | C25H41NO3 | 1000 | [M-H]- | 402.30096 | 17.36 |
| Neohancoside D | C23H32O15 |  | [M-H]-, [M+Cl]- | 547.16664 | 6.67 |
| Oritin-4beta-ol | C15H14O6 | 983.7 | [M-H]- | 289.07187 | 6.65 |
| Pantheric Acid C | C11H18O4 | 1000 | [M-H]- | 213.11342 | 9.51 |
| Plasmodiophorol C | C18H32O5 | 977.5 | [M-H]- | 327.21797 | 10.21 |
| propyl 2-(1,3-dimethyl-2,6-dioxopurin-7-yl)acetate | C12H16N4O4 | 957 | [M-H]- | 279.10883 | 5.01 |
| Quercetin 3-galactoside-7-glucosyl-(1->4)-rhamnoside | C33H40O21 | 935.4 | [M-H]- | 771.19919 | 7.18 |
| Quinoline-2,6-diol | C9H7NO2 | 916.6 | [M-H]- | 160.04054 | 6.52 |
| Siegesbeckin E | C18H34O5 | 966.5 | [M-H]- | 329.23359 | 11.56 |
| Siegesbeckin F | C18H34O5 | 1000 | [M-H]- | 329.23346 | 12.07 |
| Tetranor-PGF1alpha | C16H28O5 | 1000 | [M-H]- | 299.18699 | 10.75 |
| Thysanone | C14H12O6 | 777.9 | [M-H]- | 275.05617 | 13.21 |
| Ustilic acid A | C16H32O4 | 1000 | [M-H]- | 287.22305 | 12.05 |
| Villosone | C23H18O8 | 909.6 | [M-H]- | 421.09266 | 11.36 |
| .alpha.-Tocopheryl acetate | C31H52O3 | 850.4 | [M+H]+ | 473.39869 | 18.61 |
| 13-Keto-9Z,11E-octadecadienoic acid | C18H30O3 | 703.4 | [M+H]+ | 295.22692 | 14.12 |
| 13Z-Docosenamide | C22H43NO | 871.1 | [M+H]+ | 338.34173 | 17.2 |
| 1H-Inden-2-amine, 2,3-dihydro-N-methyl- | C10H13N | 811 | [M+H]+ | 148.11224 | 1.17 |
| 2-(4-methoxyphenyl)-1H-indole | C15H13NO | 688.6 | [M+H]+ | 224.10685 | 6.96 |
| 2-methoxy-N-methyl-N-[(2-phenoxypyridin-3-yl)methyl]acetamide | C16H18N2O3 | 695.2 | [M+H]+ | 287.13912 | 6 |
| 2'-O-Methyladenosine | C11H15N5O4 | 793.8 | [M+H]+ | 282.12014 | 1.2 |
| 3-Cyclopentene-1-octanoic acid, 2-(3-hydroxy-1-penten-1-yl)-5-oxo- | C18H28O4 | 780.8 | [M+H]+ | 309.20624 | 11.09 |
| 3-Indoleacrylic acid | C11H9NO2 | 686.1 | [M+H]+ | 188.07051 | 4.63 |
| 4-formyl Indole | C9H7NO | 877.7 | [M+H]+ | 146.06015 | 4.63 |
| 6-Methylquinoline | C10H9N | 803.2 | [M+H]+ | 144.08087 | 8.52 |
| 8(9)-Epoxy-5Z,11Z,14Z-eicosatrienoic acid | C20H32O3 | 899 | [M+H]+ | 321.24264 | 14.05 |
| 9-OxoODE | C18H30O3 | 863.6 | [M+H]+ | 295.22666 | 14.27 |
| 9(S)-HpODE | C18H32O4 | 698.7 | [M+H-H2O]+, [M+H]+ | 295.22676 | 10.18 |
| ADENINE | C5H5N5 | 953.2 | [M+H]+ | 136.06194 | 0.59 |
| Ala Gly Leu Val Ser | C19H35N5O7 | 714.2 | [M+H]+ | 446.26128 | 5.72 |
| Ala Val Leu | C14H27N3O4 | 713.5 | [M+H]+ | 302.20756 | 5.57 |
| Avobenzone | C20H22O3 | 956 | [M+H]+ | 311.164 | 16.02 |
| Bis(2-ethylhexyl) adipate | C22H42O4 | 865.9 | [M+H]+, [M+Na]+ | 371.31553 | 17.99 |
| Bis(3,5,5-trimethylhexyl) phthalate | C26H42O4 | 803.9 | [M+H]+ | 419.31562 | 18.2 |
| Bis(p-methylbenzylidene)sorbitol | C22H26O6 | 992.1 | [M+H]+ | 387.18031 | 11.35 |
| Cer 18:1;O2/16:0;O | C34H67NO4 | 502.5 | [M+H]+ | 554.51445 | 17.83 |
| CETRIMONIUM | C19H41N | 948.8 | [M+H]+ | 284.33142 | 12.36 |
| Cetylpyridinium | C21H37N | 961.9 | [M+H]+ | 304.29983 | 12.52 |
| Cyclazadone | C12H12N2O2 | 790.2 | [M+H]+ | 217.09718 | 5.92 |
| Di-N-octyl phthalate | C24H38O4 | 981.8 | [M+Na]+ | 413.26618 | 17.98 |
| Di(2-ethylhexyl) phthalate | C24H38O4 | 996.5 | [M+H]+ | 391.28419 | 17.99 |
| Di(3,7-dimethyl-1-octyl) phthalate | C28H46O4 | 942.8 | [M+H]+ | 447.34683 | 19.33 |
| Epicatechin | C15H14O6 | 839.7 | [M+H]+ | 291.0865 | 6.55 |
| Ergothioneine | C9H15N3O2S | 768.8 | [M+H]+ | 230.09617 | 5.57 |
| Erythrodiol | C30H50O2 | 855.1 | [M+H-H2O]+, [M+H]+, [M+Na]+ | 425.37668 | 18.64 |
| Ethyl {4-[(2E)-3-phenyl-2-propenoyl]phenyl}carbamate | C18H17NO3 | 841.4 | [M+H]+ | 296.12814 | 10.91 |
| ethyl 2-[3-(azepan-1-ylsulfonyl)-2-oxopyridin-1-yl]acetate | C15H22N2O5S | 848.7 | [M+H]+ | 343.13158 | 19.67 |
| Gamma-Glu-Leu | C11H20N2O5 | 919.1 | [M+H]+ | 261.14445 | 5.78 |
| Gln Gly Ile | C13H24N4O5 | 631.6 | [M+H]+ | 317.18211 | 0.65 |
| Gln Val Ile | C16H30N4O5 | 887 | [M+H]+ | 359.22942 | 5.47 |
| Gln Val Leu | C16H30N4O5 | 629.3 | [M+H]+ | 359.22869 | 5.82 |
| Gln-Cys | C8H15N3O4S | 784.3 | [M+H]+ | 250.08612 | 16.1 |
| Glutathione, oxidized | C20H32N6O12S2 | 725.8 | [M+H]+ | 613.16039 | 1 |
| Glycerol tricaprylate | C27H50O6 | 841.2 | [M+Na]+ | 493.34995 | 18.86 |
| Glycerophosphocholine | C8H20NO6P | 925.2 | [M+H]+ | 258.10986 | 0.64 |
| Guaiaverin | C20H18O11 | 962.3 | [M+H]+ | 435.09261 | 8.21 |
| Hesperetin dihydrochalcone | C16H16O6 | 641.5 | [M+H]+ | 305.10213 | 7.05 |
| Ile Asn Val Asp | C19H33N5O8 | 940 | [M+H]+ | 460.24093 | 5.49 |
| Ile Leu Glu | C17H31N3O6 | 931.8 | [M+H]+ | 374.22866 | 5.46 |
| Ile Phe | C15H22N2O3 | 961 | [M+H]+ | 279.1704 | 6.38 |
| Ile-Ile | C12H24N2O3 | 994.2 | [M+H]+ | 245.18606 | 5.06 |
| Isorhamnetin 3-glucoside | C22H22O12 | 904.6 | [M+H]+ | 479.11887 | 8.54 |
| Kaempferol | C15H10O6 | 950.8 | [M+H]+ | 287.05554 | 8.73 |
| Leu Ile Thr | C16H31N3O5 | 912.4 | [M+H]+ | 346.23392 | 5.36 |
| Leu-Leu | C12H24N2O3 | 989.9 | [M+H]+ | 245.18594 | 5.92 |
| Leu-Pro | C11H20N2O3 | 957.1 | [M+H]+ | 229.1547 | 0.63 |
| Methenolone | C20H30O2 | 628.9 | [M+H]+ | 303.23203 | 14.75 |
| Methyl 4-methoxycinnamate | C11H12O3 | 792.7 | [M+H]+ | 193.08592 | 6.46 |
| Muramic acid | C9H17NO7 | 630.3 | [M+H]+ | 252.10766 | 0.64 |
| Myristoyl ethanolamide | C16H33NO2 | 713.8 | [M+H]+ | 272.25845 | 14.02 |
| N-(2,5-dimethoxyphenyl)-4,5,6,7-tetrahydro-1,2-benzoxazole-3-carboxamide | C16H18N2O4 | 658.4 | [M+H]+ | 303.13394 | 5.67 |
| N-Oleoylethanolamine | C20H39NO2 | 726.7 | [M+H]+ | 326.30532 | 15.82 |
| N-Oleoylglycine | C20H37NO3 | 659.3 | [M+H]+ | 340.28494 | 15.51 |
| N-Valylphenylalanine | C14H20N2O3 | 986.3 | [M+H]+ | 265.15484 | 5.53 |
| N,N-Bis(2-hydroxyethyl)dodecanamide | C16H33NO3 | 681.4 | [M+H]+ | 288.2533 | 10.26 |
| N,N-Dimethyldodecan-1-amine | C14H31N | 649.8 | [M+H]+ | 214.25297 | 10.24 |
| N,N-Dimethyldodecylamine-N-oxide | C14H31NO | 606.2 | [M+H]+ | 230.24787 | 9.93 |
| N,N-Dimethylguanosine | C12H17N5O5 | 920.9 | [M+H]+ | 312.13105 | 5.39 |
| Norharmane | C11H8N2 | 832.1 | [M+H]+ | 169.07594 | 5.79 |
| Oleamide | C18H35NO | 608.2 | [M+H]+ | 282.27922 | 16.4 |
| Pantothenic Acid | C9H17NO5 | 790.9 | [M+H]+, [M+H-H2O]+ | 220.11804 | 4.82 |
| Pentaethylene glycol | C10H22O6 | 674.4 | [M+H]+ | 239.14879 | 5.83 |
| Phe Tyr | C18H20N2O4 | 626.1 | [M+H]+ | 329.14997 | 5.92 |
| Phe Val | C14H20N2O3 | 996.9 | [M+H]+ | 265.15476 | 4.92 |
| Phthalic anhydride | C8H4O3 | 829.3 | [M+H]+ | 149.02347 | 17.98 |
| Pro Thr Leu | C15H27N3O5 | 964.3 | [M+H]+ | 330.20223 | 5.27 |
| Pro-Phe | C14H18N2O3 | 999 | [M+H]+ | 263.13905 | 3.34 |
| Prostaglandin A1 ethyl ester | C22H36O4 | 870.4 | [M+H]+ | 365.26867 | 17.51 |
| quinaldine | C10H9N | 806.4 | [M+H]+ | 144.08073 | 6.12 |
| Ser Pro Thr | C12H21N3O6 | 659.4 | [M+H]+ | 304.14959 | 0.61 |
| Ser Val Leu | C14H27N3O5 | 908.8 | [M+H]+ | 318.20242 | 5.56 |
| SPB 18:1;O2 | C18H37NO2 | 581.3 | [M+H]+ | 300.28985 | 11.3 |
| SPB 20:0;O2 | C20H43NO2 | 475.1 | [M+H]+ | 330.33688 | 12.46 |
| Stearoyl ethanolamide | C20H41NO2 | 776 | [M+H]+ | 328.32118 | 16.87 |
| Stearoyl-L-carnitine | C25H49NO4 | 953.7 | [M+H]+ | 428.37361 | 13.36 |
| tert-butyl p-Toluate | C12H16O2 | 634.1 | [M+H]+ | 193.12239 | 10.81 |
| Thr-Val-Leu | C15H29N3O5 | 853.3 | [M+H]+ | 332.21825 | 5.58 |
| Trigonelline | C7H7NO2 | 939.4 | [M+H]+ | 138.05518 | 0.65 |
| Triphenyl phosphate | C18H15O4P | 874.9 | [M+H]+ | 327.07816 | 13.37 |
| Triphenylphosphine oxide | C18H15OP | 778.1 | [M+H]+ | 279.09323 | 10.81 |
| Tris(butoxyethyl)phosphate | C18H39O7P | 959.7 | [M+H]+ | 399.25069 | 13.85 |
| Tyr Leu | C15H22N2O4 | 979.4 | [M+H]+ | 295.16545 | 5.61 |
| Tyr-Ile | C15H22N2O4 | 868.3 | [M+H]+ | 295.16555 | 5.09 |
| Val Ala Val | C13H25N3O4 | 938.3 | [M+H]+ | 288.19219 | 5.86 |
| Val Asn Leu | C15H28N4O5 | 894.9 | [M+H]+ | 345.2136 | 5.82 |
| Val Gln Tyr | C19H28N4O6 | 849.3 | [M+H]+ | 409.20702 | 6.37 |
| Val Glu Leu | C16H29N3O6 | 883.5 | [M+H]+ | 360.2131 | 5.79 |
| Val Leu Tyr | C20H31N3O5 | 919.7 | [M+H]+ | 394.23391 | 6.29 |
| Val Phe | C14H20N2O3 | 829.4 | [M+H]+ | 265.15482 | 5.47 |
| Val Val Val | C15H29N3O4 | 819.7 | [M+H]+ | 316.22321 | 5.33 |
| Viscidulin I | C15H10O7 | 735.5 | [M+H]+ | 303.04988 | 8.32 |

**S5 Table**. **Compounds detected by GC-MS in *E.foeminea* fruit extract.**

| Name | RT [min] | Delta RI |
| --- | --- | --- |
| 1-Naphthalenamine | 13.516 | 12 |
| 2-Hydroxybutanedioic acid, 3TMS | 12.567 | 12 |
| 2-Hydroxyglutaric acid, 3TMS | 13.203 | 8 |
| 4-Hydroxy-3-methoxyphenylglycol, 3TMS | 19.527 | 14 |
| 4-Hydroxybenzaldehyde, 1MOX, 1TMS | 12.6 | 49 |
| Benzoic acid, 1TMS | 8.188 | 23 |
| Caprylic acid, 1TMS | 9.603 | 28 |
| Cuminol, 1TMS | 8.192 | 47 |
| D-(+)-Mannose, 1MOX, 5TMS | 18.207 | 3 |
| D-(+)-Xylose, 1MOX, 4TMS | 16.24 | 32 |
| D-Chiro-inositol, 6TMS | 18.913 | 27 |
| D-Psicofuranose isomer 1, 5TMS | 16.997 | 39 |
| Dihydroxyacetone, 2TMS | 8.17 | 12 |
| Fructose isomer 1, 5TMS | 17.019 | 50 |
| Galactose isomer 2, 1MOX, 5TMS | 18.52 | 8 |
| Glucitol, 6TMS | 18.679 | 22 |
| Glucopyranose, 5TMS | 19.26 | 48 |
| Glycerol, 3TMS | 9.068 | 16 |
| Hydroxyurea, 3TMS | 9.408 | 32 |
| Isomaltose isomer 1, 1MOX, 8TMS | 25.482 | 24 |
| Levoglucosan, 3TMS | 17.56 | 5 |
| Malic acid, 3TMS | 12.454 | 4 |
| meso-Erythritol, 4TMS | 12.748 | 42 |
| Mevalonolactone, 1TMS | 10.596 | 1 |
| Myoinositol, 6TMS | 20.476 | 10 |
| N-Acetylglucosamine isomer 1, 4TMS | 20.546 | 6 |
| Nicotinic acid, 1TMS | 9.459 | 16 |
| Pyrrole-2-carboxylic acid, 2TMS | 9.684 | 11 |
| Quinic acid, 5TMS | 17.77 | 23 |
| Ribose isomer 2, 4TMS | 14.355 | 27 |
| Scyllo-inositol, 6TMS | 19.637 | 13 |
| Shikimic acid, 4TMS | 17.456 | 25 |
| Succinic acid, 2TMS | 11.521 | 8 |
| Sucrose, 8TMS | 24.608 | 7 |
| Trehalose, 8TMS | 25.1 | 14 |
| Xylitol, 5TMS | 15.9 | 12 |


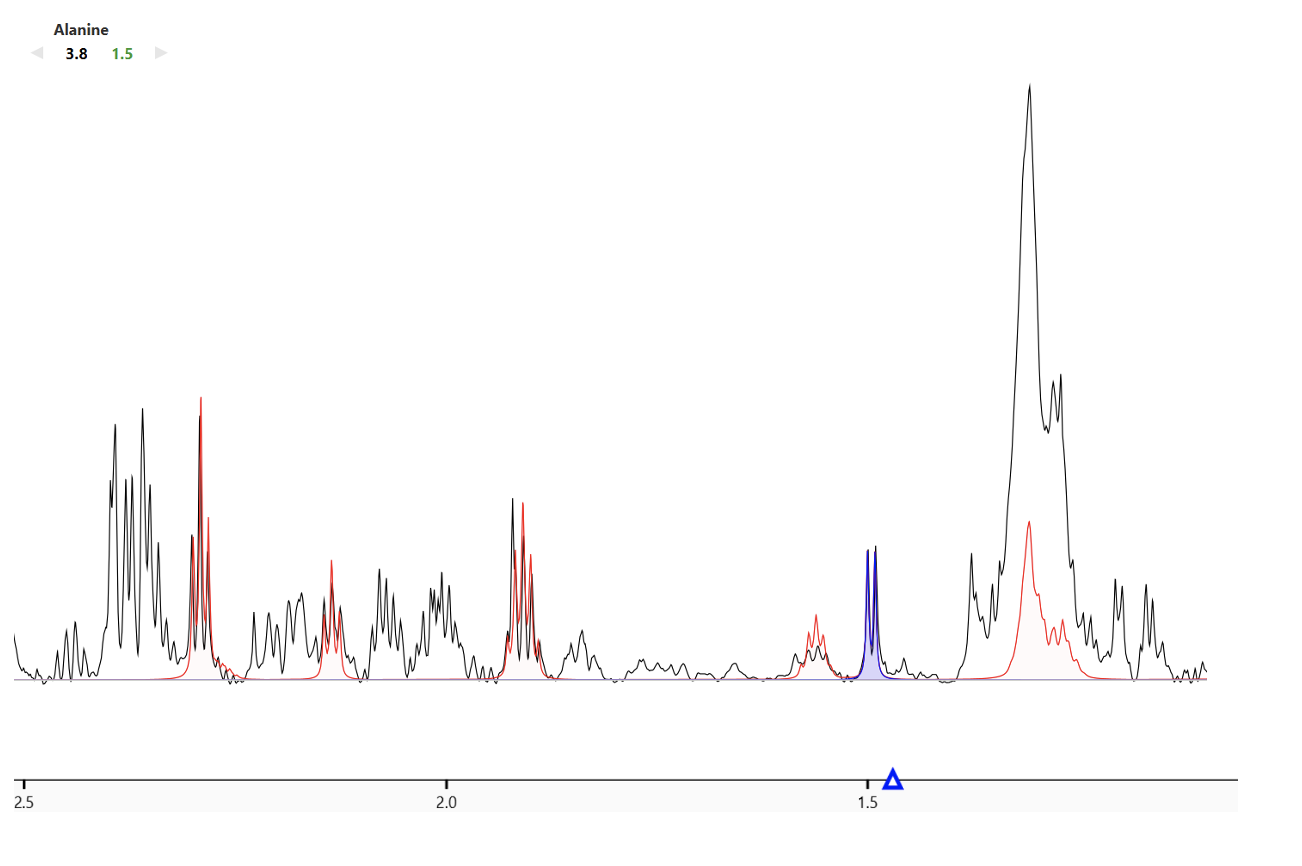


**S1 Fig**. **Screenshot of Chenomx Profiler (Chenomx Suite 9.0, Alberta, Canada) software**. The peak at 1.5 ppm can be assigned to Alanine.


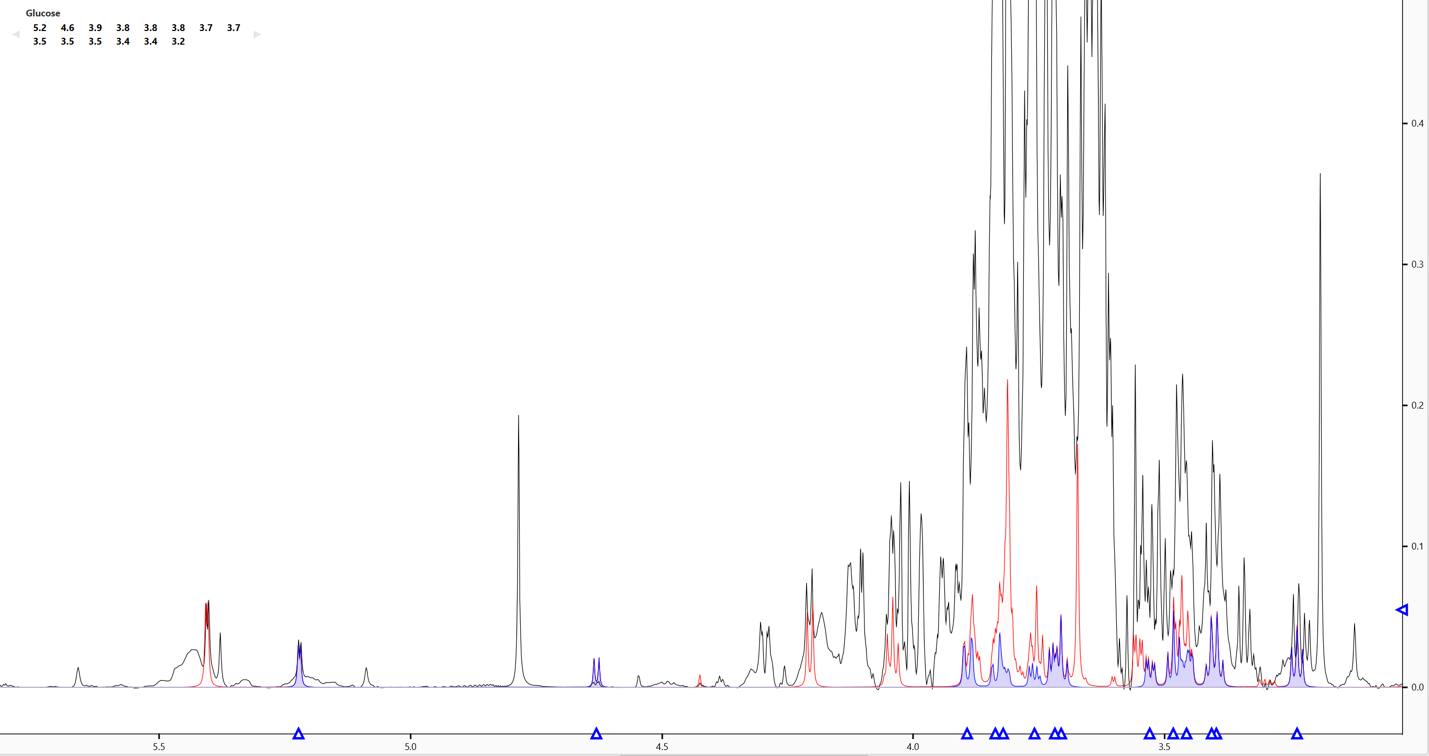


**S2 Fig**. **Screenshot of Chenomx Profiler (Chenomx Suite 9.0, Alberta, Canada) software.** The peaks from 3.2 ppm to 5.2 ppm can be assigned to glucose.


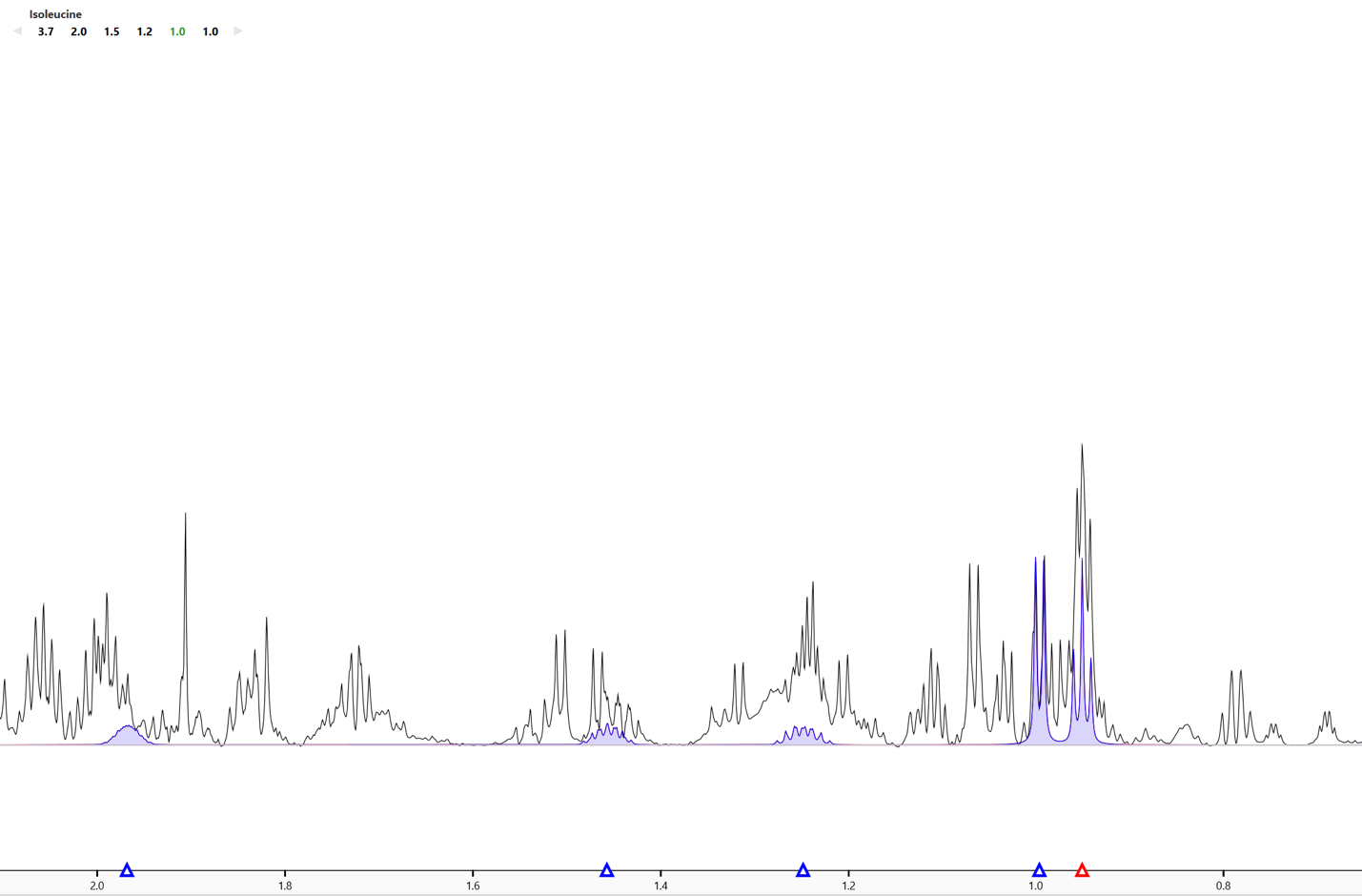


**S3 Fig**. **Screenshot of Chenomx Profiler (Chenomx Suite 9.0, Alberta, Canada) software window.** The peak at 1 ppm can be assigned to Isoleucine.


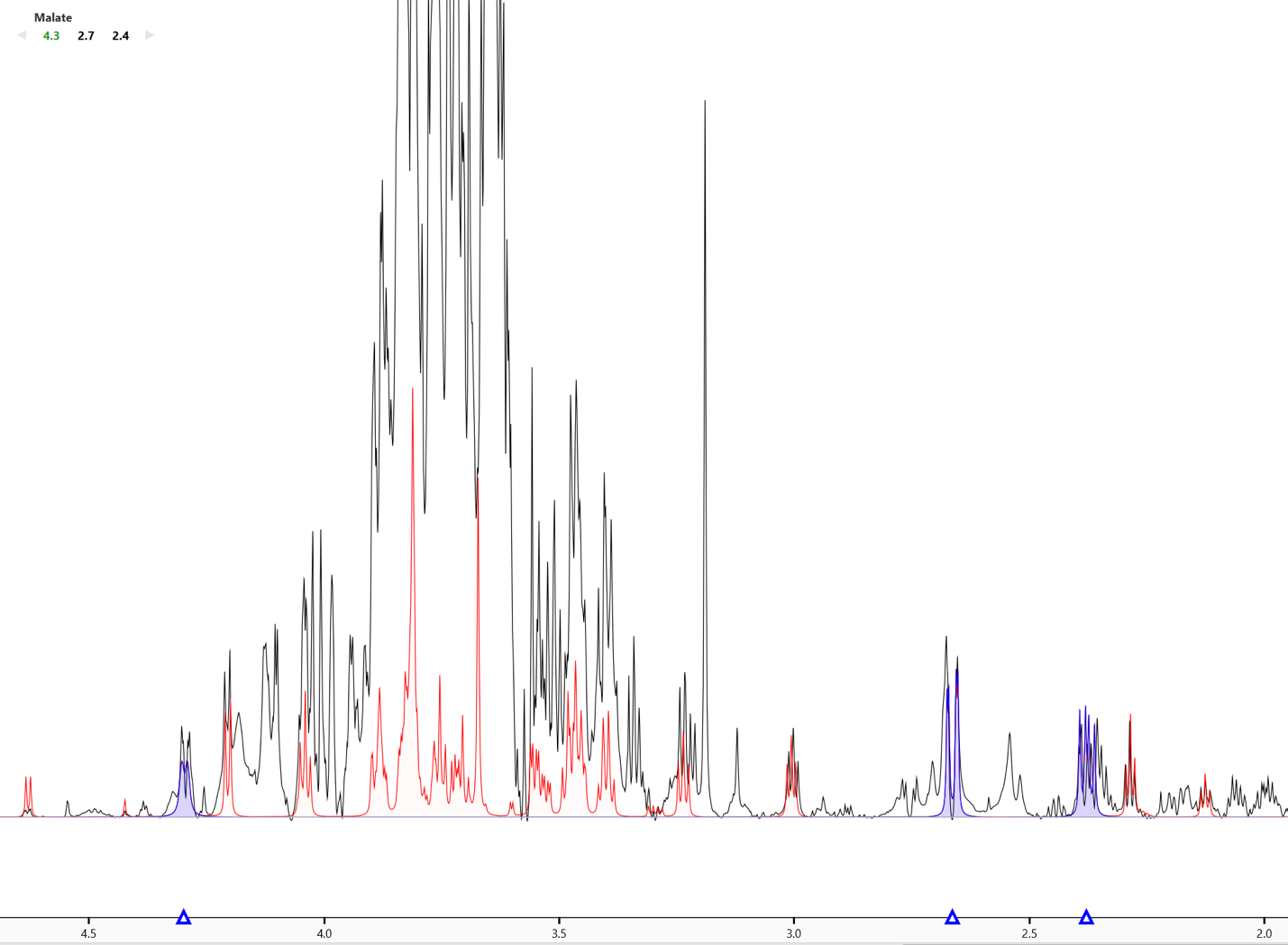


**S4 Fig**. **Screenshot of Chenomx Profiler (Chenomx Suite 9.0, Alberta, Canada) software window**. The peaks at 4.3 ppm and 2.7 ppm can be assigned to Malate.


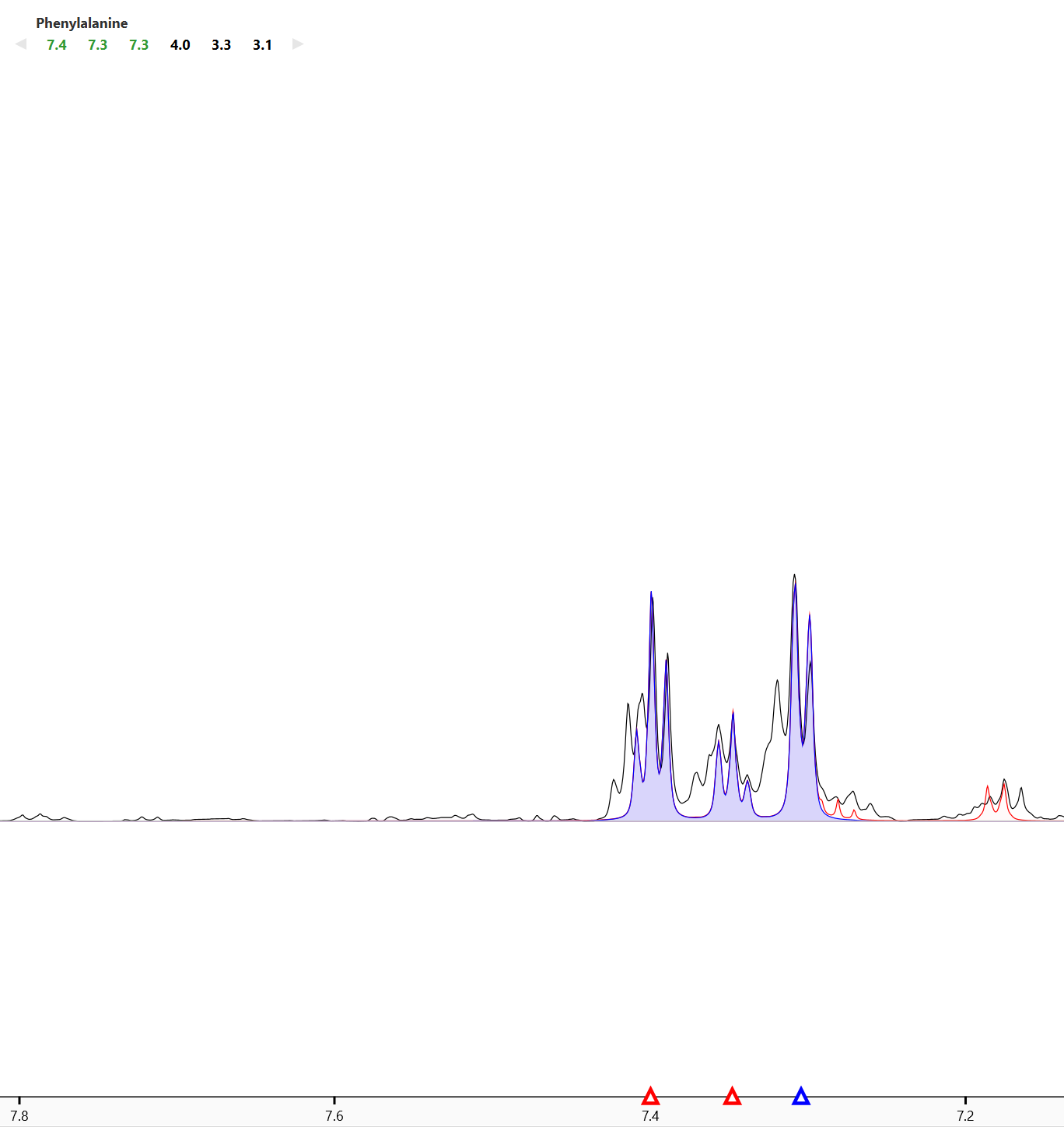


**S5 Fig**. **Screenshot of Chenomx Profiler (Chenomx Suite 9.0, Alberta, Canada) software window**. The peaks at 7.4 ppm and 7.3 ppm can be assigned to phenylalanine.


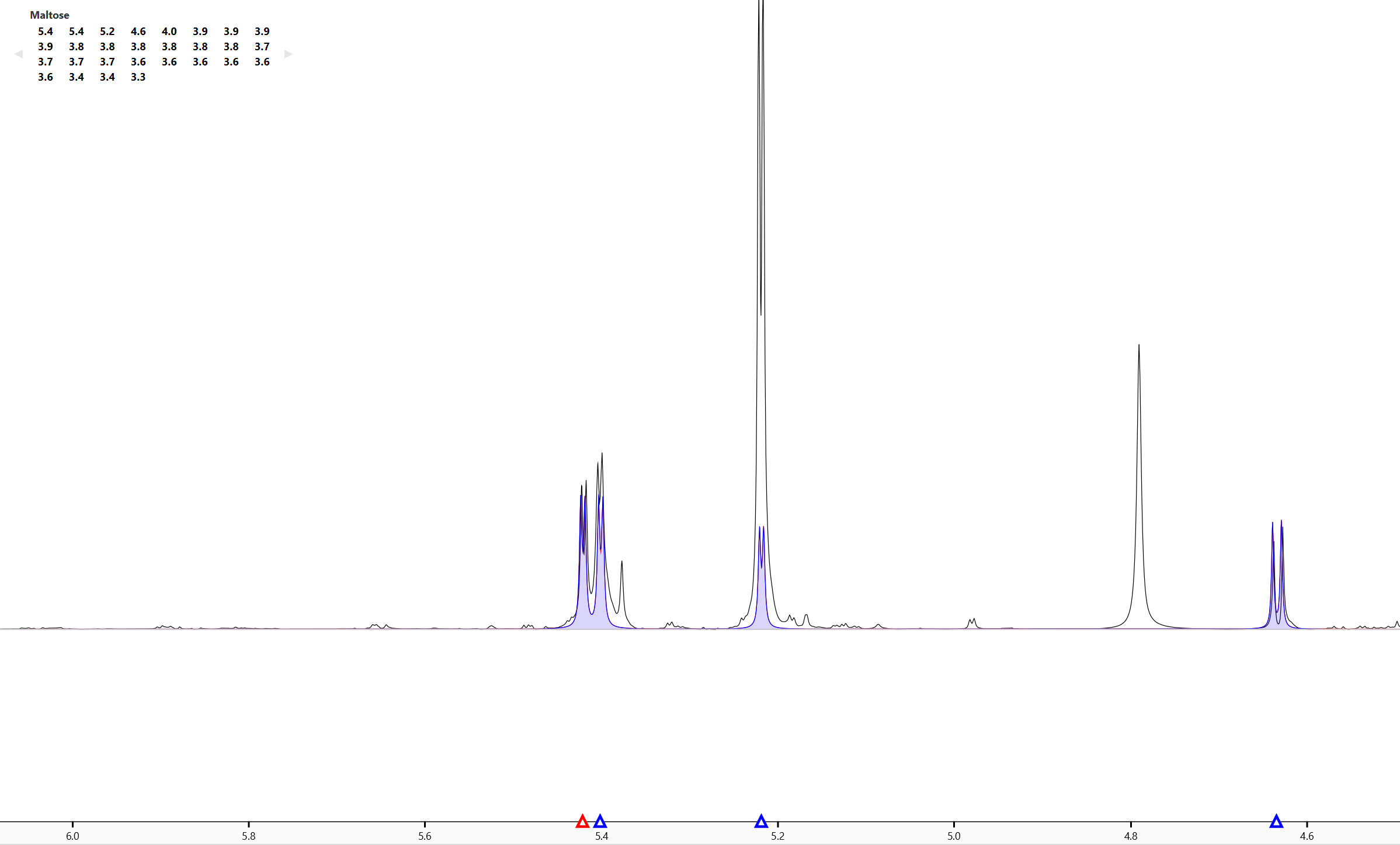


**S6 Fig**. **Screenshot of Chenomx Profiler (Chenomx Suite 9.0, Alberta, Canada) software window.** The peaks at 5.4 ppm and 4.6 ppm can be assigned to Maltose.

**S6 Table: Tentative NMR assignments of selected metabolites detected in *Ephedra foeminea* extract.**

| Compound | Nucleus | δ (ppm) | Multiplicity | Coupling constant J (Hz) | Assignment source |
| --- | --- | --- | --- | --- | --- |
| Alanine | ¹H | 1.5 | d | 7.5 | Chenomx Profiler |
| Glucose (α anomeric proton) | ¹H | 5.2 | d | 3.9 | Chenomx Profiler |
| Isoleucine | ¹H | 1 | d | 7.1 | Chenomx Profiler |
| Malate | ¹H | 2.7 | dd | 16, 3 | Chenomx Profiler |
| Phenylalanine | ¹H | 7.4-7.3 | multiplet | - | Chenomx Profiler |
| Maltose | ¹H | 5.4  4.6 | dd  d | 8 | Chenomx Profiler |

The table reports the nucleus, chemical shift (δ, ppm), multiplicity, coupling constant (J, Hz), and assignment source for each identified signal. Chemical shifts were assigned using Chenomx Profiler.


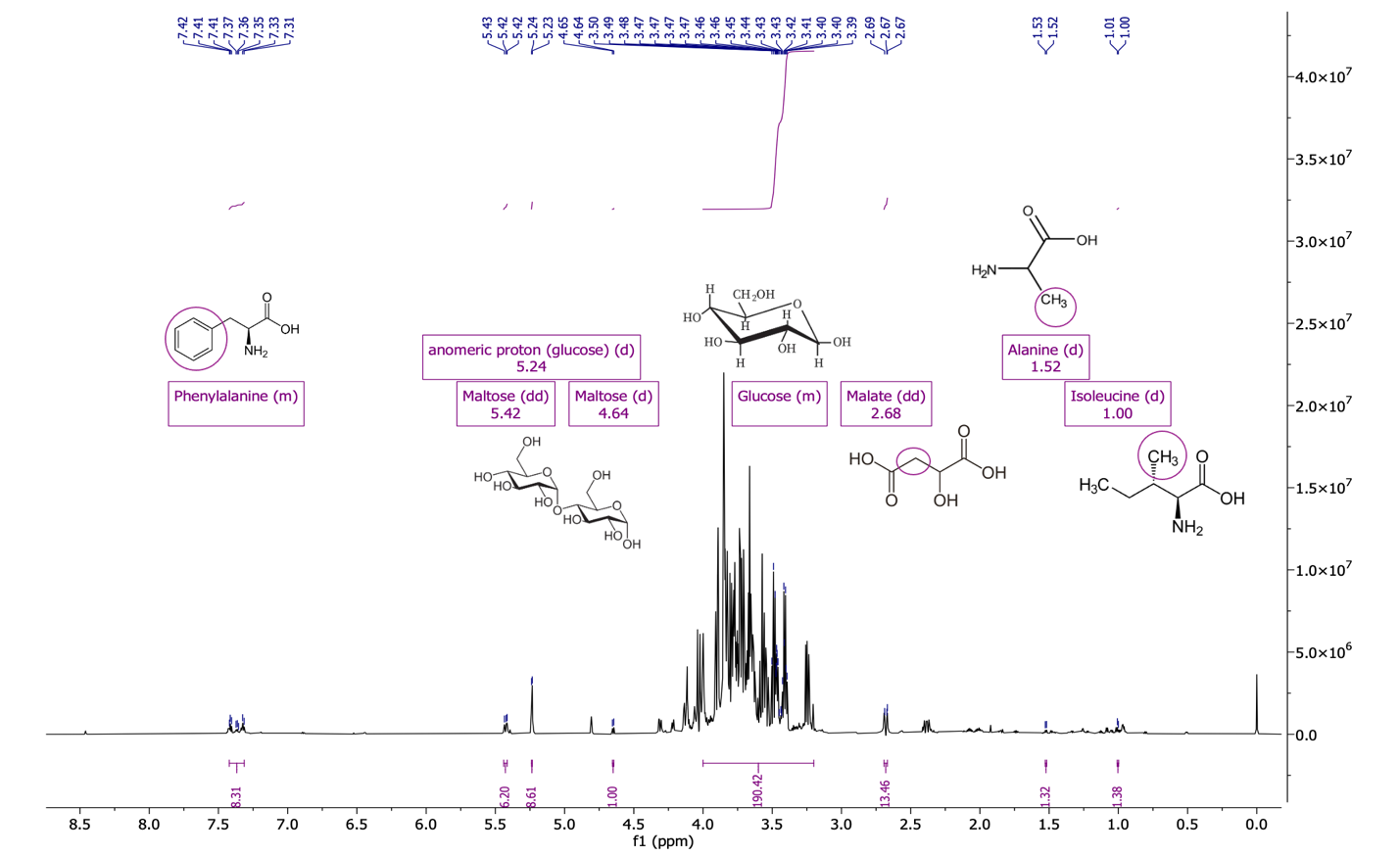


**S7 Fig. Full NMR spectra of *E. foeminea****.* The spectrum was processed and analyzed using MestReNova (v14.2.1, Mestrelab Research) and selected peaks were tentatively assigned to representative metabolites using Chenomx Profiler (Chenomx Suite 9.0, Alberta, Canada) based on chemical shift information. Corresponding compound structures and peak annotations are shown, based on the ones reported in Figs. S1–S6. This spectrum provides a chemical fingerprint of the extract, highlighting major metabolite classes.

**References**

[1] N. Mohamadi, F. Sharififar, M. Pournamdari, and M. Ansari, “A Review on Biosynthesis, Analytical Techniques, and Pharmacological Activities of Trigonelline as a Plant Alkaloid,” *J Diet Suppl*, vol. 15, no. 2, pp. 207–222, Mar. 2018, doi: 10.1080/19390211.2017.1329244.

[2] Y. Liang *et al.*, “The neuroprotective and antidiabetic effects of trigonelline: A review of signaling pathways and molecular mechanisms,” Mar. 01, 2023, *Elsevier B.V.* doi: 10.1016/j.biochi.2022.10.009.

[3] C. R. Sahoo, S. K. Paidesetty, and R. N. Padhy, “Norharmane as a potential chemical entity for development of anticancer drugs,” Jan. 15, 2019, *Elsevier Masson s.r.l.* doi: 10.1016/j.ejmech.2018.11.024.

[4] P. P. Tshikhudo *et al.*, “Anticancer Potential of β-Carboline Alkaloids: An Updated Mechanistic Overview,” Feb. 01, 2024, *John Wiley and Sons Inc*. doi: 10.1002/cbdv.202301263.

[5] G. Almaguer *et al.*, “Anticancer potential of (−)-epicatechin in a triple-negative mammary gland model,” *Journal of Pharmacy and Pharmacology*, vol. 73, no. 12, pp. 1675–1682, Dec. 2021, doi: 10.1093/jpp/rgab133.

[6] J. Shay, H. A. Elbaz, I. Lee, S. P. Zielske, M. H. Malek, and M. Hüttemann, “Molecular mechanisms and therapeutic effects of (-)-epicatechin and other polyphenols in cancer, inflammation, diabetes, and neurodegeneration,” 2015, *Hindawi Publishing Corporation*. doi: 10.1155/2015/181260.

[7] A. Budzianowska, E. Totoń, A. Romaniuk-Drapała, M. Kikowska, and J. Budzianowski, “Cytotoxic Effect of Phenylethanoid Glycosides Isolated from Plantago lanceolata L.,” *Life*, vol. 13, no. 2, Feb. 2023, doi: 10.3390/life13020556.

[8] A. Dehnoee, R. J. Kalbasi, S. Tavakoli, M. M. Zangeneh, A. Zangeneh, and M.-R. Delnavazi, “Anticancer potential of furanocoumarins and flavonoids of Heracleum persicum fruit,” Jun. 29, 2023. doi: 10.21203/rs.3.rs-3073212/v1.

[9] R. Tundis, M. R. Loizzo, M. Bonesi, F. Menichini, G. A. Statti, and F. Menichini, “In vitro Cytotoxic Activity of Salsola oppositifolia Desf. (Amaranthaceae) in a Panel of Tumour Cell Lines,” 2008. [Online]. Available: http://www.znaturforsch.com

[10] O. M. Tsivileva and O. V. Koftin, “Fungal coumarins: biotechnological and pharmaceutical aspects,” in *Studies in Natural Products Chemistry*, vol. 78, Elsevier B.V., 2023, pp. 441–479. doi: 10.1016/B978-0-323-91253-2.00010-8.

[11] S. Kaur, P. Mendonca, and K. F. A. Soliman, “The Anticancer Effects and Therapeutic Potential of Kaempferol in Triple-Negative Breast Cancer,” Aug. 01, 2024, *Multidisciplinary Digital Publishing Institute (MDPI)*. doi: 10.3390/nu16152392.

[12] E. F. de Morais *et al.*, “The Anticancer Potential of Kaempferol: A Systematic Review Based on In Vitro Studies,” Feb. 01, 2024, *Multidisciplinary Digital Publishing Institute (MDPI)*. doi: 10.3390/cancers16030585.

[13] M. Shahbaz *et al.*, “Anticancer, antioxidant, ameliorative and therapeutic properties of kaempferol,” 2023, *Taylor and Francis Ltd.* doi: 10.1080/10942912.2023.2205040.

[14] J. Mouton and F. Van Der Kooy, “Unlocking the full (medicinal) potential of Artemisia annua: A LC-MS and NMR investigation of the tea infusion.”

[15] “10.1515_znc-2012-5-606”.

[16] A. Tamaki, T. Ide, and H. Otsuka, “Phenolic glycosides from the leaves of alangium platanifolium var. platanifolium,” *J Nat Prod*, vol. 63, no. 10, pp. 1417–1419, 2000, doi: 10.1021/np000119l.

[17] L. Han *et al.*, “Ethnobotany, phytochemistry and pharmacological effects of plants in genus cynanchum linn. (Asclepiadaceae),” 2018, *MDPI AG*. doi: 10.3390/molecules23051194.

[18] National Center for Biotechnology Information (2024), “PubChem Compound Summary for CID 133556528, Neohancoside D, Sibiricose A1.” Accessed: Nov. 28, 2024. [Online]. Available: https://pubchem.ncbi.nlm.nih.gov/compound/Neohancoside-D_-Sibiricose-A1.

[19] P. Mittal, V. Gupta, G. Kaur, A. K. Garg, and A. Singh, “PHYTOCHEMISTRY AND PHARMACOLOGICAL ACTIVITIES OF PSIDIUM GUAJAVA: A REVIEW,” vol. 1, no. 9, pp. 9–19, 2010, [Online]. Available: www.ijpsr.com

[20] S. Li, R. Wang, X. Hu, C. Li, and L. Wang, “Bio-affinity ultra-filtration combined with HPLC-ESI-qTOF-MS/MS for screening potential α-glucosidase inhibitors from Cerasus humilis (Bge.) Sok. leaf-tea and in silico analysis,” *Food Chem*, vol. 373, Mar. 2022, doi: 10.1016/j.foodchem.2021.131528.

[21] S. Narwal, S. Kumar, and P. K. Verma, “Synthesis and therapeutic potential of quinoline derivatives,” May 01, 2017, *Springer Netherlands*. doi: 10.1007/s11164-016-2794-2.

[22] V. A. Wahyudi, A. C. Nisya, H. A. Manshur, A. Husna, and Syarpin, “Optimisation of corn silk tea production, and its antioxidant profile,” *Int Food Res J*, vol. 31, no. 3, pp. 670–680, Jun. 2024, doi: 10.47836/ifrj.31.3.12.

[23] C. R. Quijia and M. Chorilli, “Piperine for treating breast cancer: A review of molecular mechanisms, combination with anticancer drugs, and nanosystems,” Jan. 01, 2022, *John Wiley and Sons Ltd*. doi: 10.1002/ptr.7291.

[24] S. Mitra *et al.*, “Anticancer Applications and Pharmacological Properties of Piperidine and Piperine: A Comprehensive Review on Molecular Mechanisms and Therapeutic Perspectives,” Jan. 07, 2022, *Frontiers Media S.A.* doi: 10.3389/fphar.2021.772418.

[25] J. L. Ríos, G. R. Schinella, and I. Moragrega, “Phenolics as GABAA Receptor Ligands: An Updated Review,” Mar. 01, 2022, *MDPI*. doi: 10.3390/molecules27061770.

[26] D. Iacopetta *et al.*, “An Update on Recent Studies Focusing on the Antioxidant Properties of Salvia Species,” Dec. 01, 2023, *Multidisciplinary Digital Publishing Institute (MDPI)*. doi: 10.3390/antiox12122106.

[27] E. B. Khalid, E.-M. E.-K. Ayman, H. Rahman, G. Abdelkarim, and A. Najda, “Natural products against cancer angiogenesis,” *Tumor Biology*, vol. 37, no. 11, pp. 14513–14536, 2016, doi: 10.1007/s13277-016-5364-8.

[28] A. López-Jiménez, M. García-Caballero, M. Á. Medina, and A. R. Quesada, “Anti-angiogenic properties of carnosol and carnosic acid, two major dietary compounds from rosemary,” *Eur J Nutr*, vol. 52, no. 1, pp. 85–95, Feb. 2013, doi: 10.1007/s00394-011-0289-x.
